# Apatinib inhibits synovial sarcoma progression and angiogenesis via VEGFR2-mediated AKT/FOXO3A and ERK1/2/FOXM1 signaling pathways

**DOI:** 10.1038/s41420-026-03188-7

**Published:** 2026-06-03

**Authors:** Ruijie Liu, Fengzhen Zhang, Kai Shi, Wenhao Wang, Xuewen Li, Shaobo Lu, Bowen Chen, Dongjin Wu, Changliang Peng

**Affiliations:** Department of Spinal Surgery, The Second Qilu Hospital of Shandong University, Jinan, China

**Keywords:** Sarcoma, Targeted therapies, Tumour angiogenesis

## Abstract

Synovial sarcoma (SS) is a highly aggressive soft-tissue malignancy with limited therapeutic options for advanced disease. Here, we report that apatinib, a selective VEGFR2 tyrosine kinase inhibitor, exerts potent antitumor and anti-angiogenic activity in SS. In SS cell lines, apatinib reduced cell viability, induced G1 phase arrest, and triggered both apoptosis and autophagy. Apatinib further suppressed the metastatic phenotype by inhibiting cell migration, invasion, and EMT, while simultaneously reducing VEGF expression and impairing endothelial tube formation. Mechanistically, apatinib suppressed the VEGFR2/AKT/FOXO3A and ERK1/2/FOXM1 axes in tumor cells, thereby restraining proliferation and angiogenic signaling, and directly impaired endothelial function via the VEGFR2/AKT/eNOS/NO pathway in HUVECs. In vivo, apatinib markedly suppressed xenograft tumor growth and modulated the corresponding signaling pathways. Collectively, our findings identify apatinib as a promising therapeutic agent that disrupts interconnected survival and angiogenic networks in SS.

## Introduction

Synovial sarcoma (SS) is a rare, highly aggressive soft-tissue malignancy affecting mainly adolescents and young adults and most commonly arising in the extremities [1]. Despite advances in multimodal therapy, SS frequently recurs and metastasizes, and prognosis is poor once the disease becomes unresectable or metastatic [2–4]. Molecularly, SS is defined by the pathognomonic t(X;18)(p11;q11) translocation, generating SS18–SSX fusion oncogenes that drive tumor initiation and maintenance [5]. For localized disease, complete resection with negative margins combined with radiotherapy is standard [6]. However, outcomes for metastatic SS remain unsatisfactory despite anthracycline-based chemotherapy, trabectedin, and multi-targeted tyrosine kinase inhibitors (TKIs) [7]. Among anti-angiogenic TKIs, pazopanib improved progression-free survival in the phase III PALETTE trial, but objective responses remain limited and resistance frequently develops, highlighting the need to identify novel angiogenesis-related targets and key signaling pathways in SS [7, 8].

Angiogenesis is essential for tumor proliferation, invasion, and metastasis [9], and the VEGF-VEGFR2 axis is the dominant driver of endothelial activation and vascular tube formation [10]. In SS, elevated VEGF expression correlates with adverse outcomes [11], and the SS18-SSX fusion modulates VEGF signaling [12], supporting VEGF pathway targeting in this disease. VEGF-activated VEGFR2 engages the PI3K/AKT signaling pathway, promoting phosphorylation of endothelial nitric oxide synthase (eNOS) and nitric oxide (NO)-mediated pro-angiogenic responses [13, 14]. These observations position VEGFR2 signaling as a rational target to suppress both tumor angiogenesis and metastatic capacity in SS.

Apatinib is an orally bioavailable, highly selective VEGFR2 TKI [15] that has demonstrated clinical activity across multiple solid tumors, including gastric cancer [16], hepatocellular carcinoma [17], and lung cancer [18]. Emerging evidence suggests benefit in advanced sarcomas [19], with a recent case series in advanced SS showing promising results [20]. However, the molecular mechanisms underlying apatinib efficacy in SS remain incompletely defined. Therefore, elucidating its antitumor effects and downstream signaling consequences in SS represents a priority for rational clinical development.

Beyond endothelial cells, VEGFR2 activation drives pro-survival and pro-proliferative programs in tumor cells via the PI3K/AKT and MAPK/ERK pathways [21]. These pathways converge on transcriptional regulators governing cell-cycle progression and angiogenesis. AKT phosphorylates FOXO3A to promote its nuclear export, suppressing p27^Kip1 transcription and facilitating G1/S transition [22–24]. In parallel, ERK1/2 sustains FOXM1 expression and activity, driving both proliferation and pro-angiogenic gene expression (including VEGF), thereby establishing a positive feedback loop [25, 26]. Together, these mechanisms indicate that VEGFR2 blockade can simultaneously disrupt tumor angiogenesis and suppress tumor cell proliferation and metastasis via the AKT/FOXO3A and ERK1/2/FOXM1 pathways.

In this study, we systematically investigated the effects of apatinib in SS. We demonstrate that apatinib suppresses tumor growth by inhibiting proliferation, inducing G1 arrest, and activating apoptosis and autophagy. Apatinib also attenuates migration, invasion, and EMT. Mechanistically, apatinib targets the VEGFR2/AKT/FOXO3A and VEGFR2/ERK1/2/FOXM1 signaling axes to inhibit cell-cycle progression and angiogenic gene expression, while directly impairing endothelial function via disruption of the VEGFR2/AKT/eNOS/NO pathway. These findings establish a multilayered mechanistic framework supporting clinical development of VEGFR2-targeted therapy in SS.

## Results

### Apatinib suppresses viability and induces cell death in SS cells

**Fig. 1:**
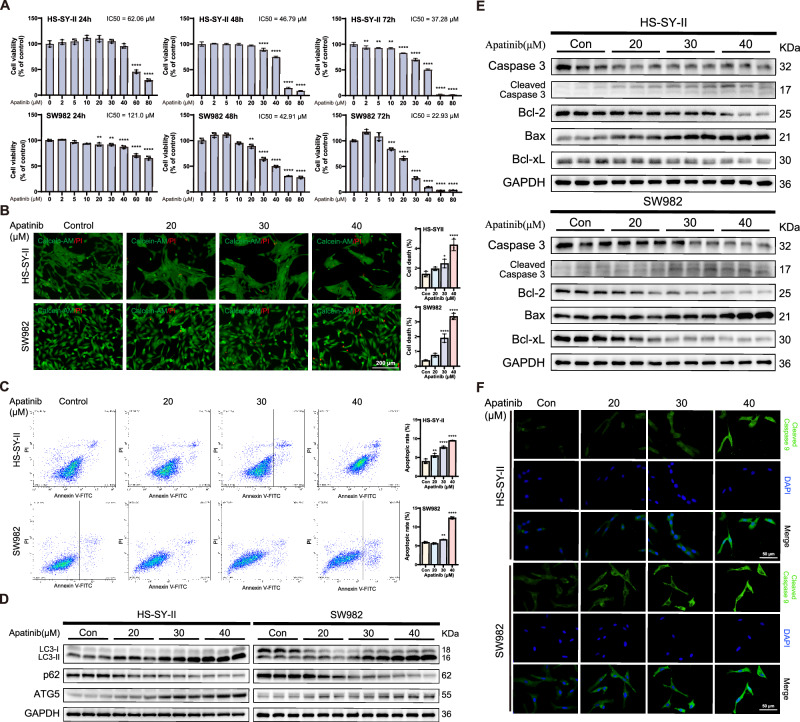
Apatinib reduces cell viability and induces cell death in SS cells. **A** Cell viability of HS-SY-II and SW982 cells treated with indicated concentrations of apatinib for 24, 48, and 72 h, assessed by CCK-8 assay. **B** Representative fluorescence images and quantification of PI-positive cells in HS-SY-II and SW982 after apatinib treatment, assessed by Calcein-AM/PI staining. Live cells exhibit green fluorescence, whereas dead cells display red fluorescence with condensed or fragmented chromatin. Scale bar = 200 μm. **C** Flow cytometric analysis of apoptosis by Annexin V/PI staining in HS-SY-II and SW982 cells after apatinib treatment and quantification of apoptotic cells. **D** Western blot analysis of autophagy markers LC3, p62, and ATG5 in apatinib-treated cells. **E** Western blot analysis of the apoptosis markers cleaved caspase-3, total caspase-3, Bcl-2, Bcl-xL, and Bax in apatinib-treated cells. **F** Representative immunofluorescence images of cleaved caspase-9 (green) and DAPI (blue) in HS-SY-II and SW982 cells after apatinib treatment. Scale bar = 50 μm. Data are presented as mean ± SD (*n* = 3 biological replicates). Statistical significance was determined by one-way ANOVA. *p < 0.05, **p < 0.01, ***p < 0.001, ****p < 0.0001.

To evaluate the cytotoxic effects of apatinib on SS cells, we treated HS-SY-II and SW982 cell lines with apatinib (0–80 μM) for 24, 48, and 72 h. CCK-8 viability assays showed that apatinib dose-dependently and time-dependently reduced cell viability in both cell lines (Fig. 1A). Based on these dose-response profiles, subsequent mechanistic experiments employed apatinib concentrations of 20, 30, and 40 μM for 48 h.

To determine whether diminished viability reflected cytotoxic cell death, we performed Calcein-AM/PI live/dead staining and Annexin V/PI flow cytometric analysis. Apatinib treatment concentration-dependently increased the proportion of PI-positive cells and the overall apoptotic cell fraction, confirming induction of cell death (Fig. 1B, C). To characterize the specific modes of programmed cell death engaged by apatinib, we next examined molecular markers of autophagy and apoptosis. Western blot analysis demonstrated that apatinib activated autophagy, as indicated by increased LC3-II/LC3-I ratio and ATG5 expression together with reduced p62 levels, indicating autophagic activation (Fig. 1D). Concurrently, apatinib activated the intrinsic apoptotic cascade, as reflected by elevated levels of cleaved caspase-3 and the pro-apoptotic protein Bax, coupled with downregulation of the anti-apoptotic Bcl-2 and Bcl-xL (Fig. 1E). Consistently, immunofluorescence staining revealed marked upregulation of cleaved caspase-9, the initiator caspase of the mitochondrial apoptotic pathway, in apatinib-treated cells (Fig. 1F). Collectively, these data establish that apatinib potently suppresses SS cell viability through concurrent activation of both autophagic and apoptotic cell death programs.

### Apatinib suppresses migration, invasion, EMT, and angiogenesis in SS cells

**Fig. 2:**
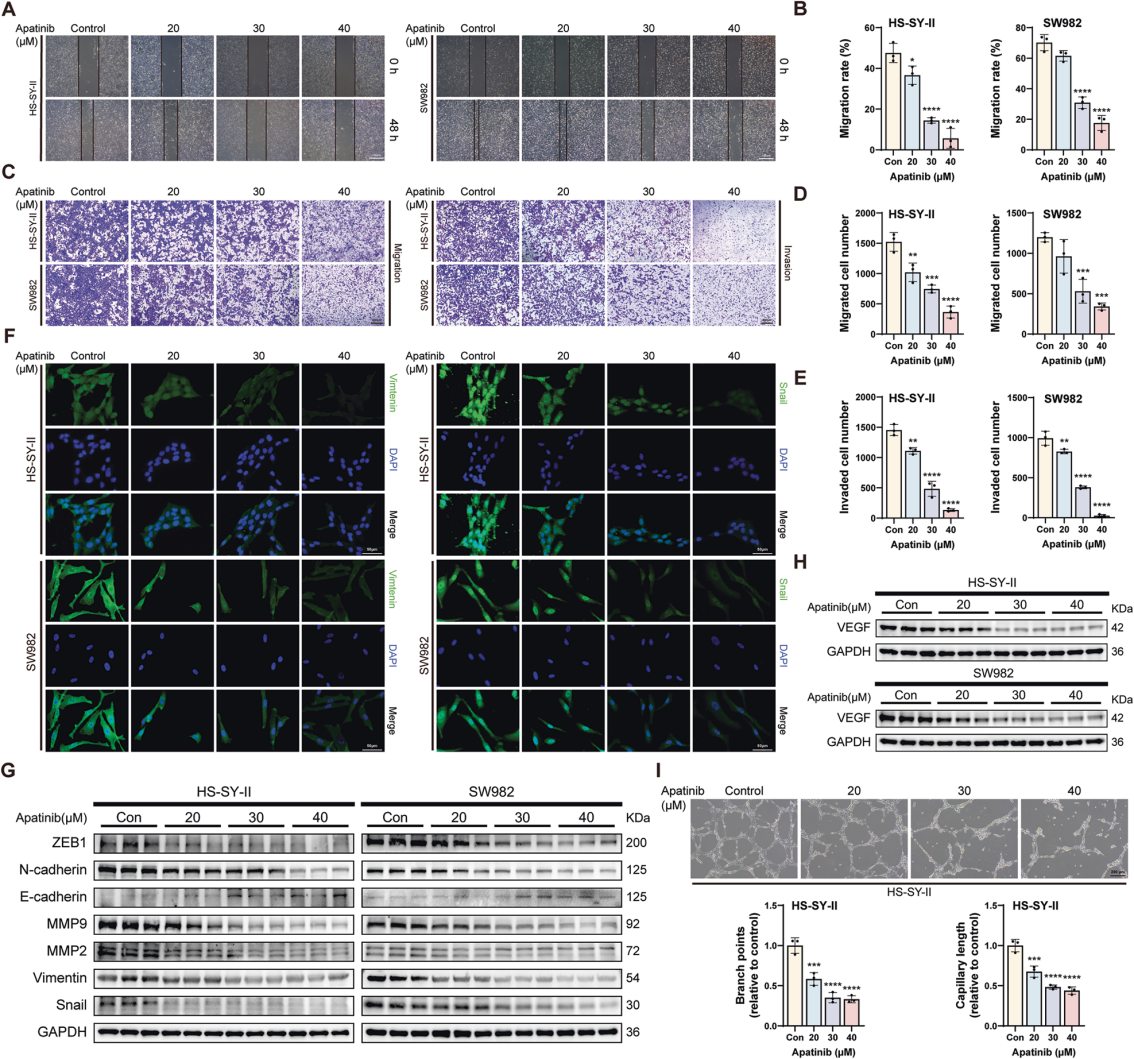
Apatinib inhibits migration, invasion, EMT, and angiogenic activity in SS cells. **A**, **B** Representative images and quantification of wound closure in HS-SY-II and SW982 cells after apatinib treatment, assessed by wound healing assay. Scale bar = 100 μm. **C**–**E** Representative images and quantification of migrated and invaded HS-SY-II and SW982 cells after apatinib treatment, assessed by Transwell migration and invasion assays. Scale bar = 200 μm. **F** Representative immunofluorescence images of Vimentin and Snail (green) and DAPI (blue) in HS-SY-II and SW982 cells after apatinib treatment. Scale bar = 50 μm. **G** Western blot analysis of EMT markers (E-cadherin, N-cadherin, Vimentin, Snail, and ZEB1) and invasion-related proteins (MMP9, MMP2) in apatinib-treated cells. **H** Western blot analysis of VEGF expression in apatinib-treated SS cells. **I** Representative images and quantification of total tube length and branch points in HUVECs cultured with conditioned medium from apatinib-treated SS cells, assessed by tube formation assay. Scale bar = 200 μm. Data are presented as mean ± SD (*n* = 3 biological replicates). Statistical significance was determined by one-way ANOVA. *p < 0.05, **p < 0.01, ***p < 0.001, ****p < 0.0001.

To investigate the effect of apatinib on the metastatic potential of SS cells, we performed functional migration and invasion assays. Wound healing assays showed that apatinib dose-dependently delayed wound closure in SS cells over 48 h, indicating impaired migration (Fig. 2A, B). Consistently, Transwell assays revealed that apatinib significantly reduced the numbers of migrating cells and invading cells in a concentration-dependent manner (Fig. 2C–E). Together, these results indicate that apatinib suppresses the migratory and invasive capacities of SS cells.

Given that epithelial-mesenchymal transition (EMT) represents a key driver of tumor metastasis, we next examined the impact of apatinib on EMT-associated molecular markers. Western blot and immunofluorescence analyses demonstrated that apatinib treatment dose-dependently upregulated the epithelial marker E-cadherin while concurrently downregulating mesenchymal markers (N-cadherin and Vimentin), EMT-inducing transcription factors (Snail and ZEB1), and matrix-remodeling proteases (MMP2 and MMP9) (Fig. 2F, G). This reciprocal modulation of epithelial and mesenchymal markers confirms that apatinib reverses the EMT phenotype in SS cells.

Because tumor-derived angiogenic factors play a critical role in sustaining metastatic dissemination by promoting neovascularization at both primary and metastatic sites, we next evaluated the effect of apatinib on angiogenesis. Western blot analysis revealed that apatinib treatment dose-dependently reduced VEGF expression in SS cells (Fig. 2H). To assess the functional consequences of this reduction, we performed endothelial tube formation assays using conditioned medium (CM) harvested from vehicle- or apatinib-treated SS cells. Strikingly, CM from apatinib-treated SS cells markedly impaired tube formation compared with vehicle CM (Fig. 2I). Collectively, these results demonstrate that apatinib suppresses metastatic phenotypes through EMT reversal and reduced pro-angiogenic signaling.

### Apatinib suppresses proliferation and induces G1 phase cell-cycle arrest

**Fig. 3:**
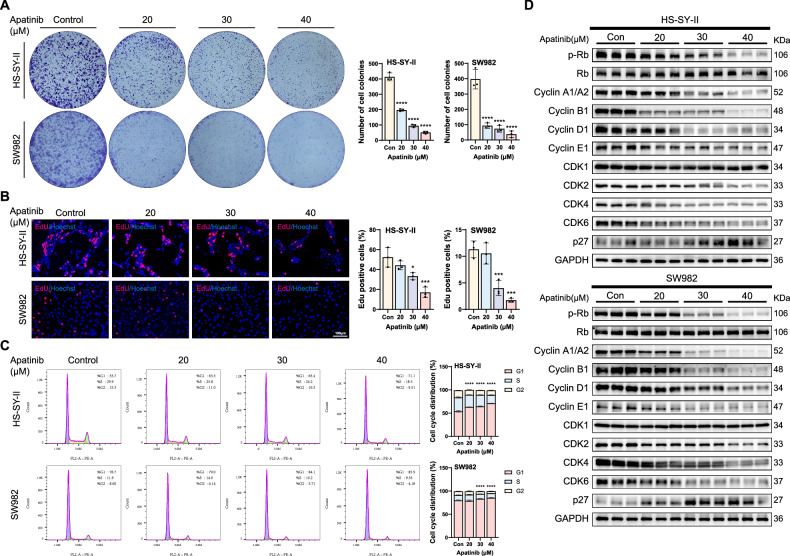
Apatinib suppresses proliferation by inducing G1 phase arrest in SS cells. **A** Representative images and quantification of colony numbers in HS-SY-II and SW982 cells after apatinib treatment, assessed by colony formation assay. **B** Representative immunofluorescence images and quantification of EdU-positive cells (EdU, red; Hoechst 33342, blue) in HS-SY-II and SW982 cells after apatinib treatment, assessed by EdU incorporation assay. Scale bar = 100 μm. **C** Representative flow cytometry plots and quantification of cell-cycle distribution (G1, S, and G2 phases) in HS-SY-II and SW982 cells after apatinib treatment. **D** Western blot analysis of cell-cycle regulators (p-Rb, Rb, cyclin A1/A2, cyclin B1, cyclin D1, cyclin E1, CDK1, CDK2, CDK4, CDK6, and p27) after apatinib treatment. Data are presented as mean ± SD (*n* = 3 biological replicates). Statistical significance was determined by one-way ANOVA. *p < 0.05, ***p < 0.001, ****p < 0.0001.

To examine the effect of apatinib on SS cell proliferation, we performed colony formation and EdU incorporation assays. Colony formation assays demonstrated that apatinib dose-dependently reduced the number of colonies formed by HS-SY-II and SW982 cells over 14 days (Fig. 3A). Similarly, EdU incorporation assays revealed that apatinib significantly reduced the proportion of EdU-positive cells (Fig. 3B), establishing that apatinib potently suppresses SS cell proliferation. To elucidate the underlying mechanism, we analyzed cell cycle distribution using flow cytometry. The results demonstrated that apatinib increased the proportion of cells in the G1 phase and reduced those in the S phase, indicating a G1/S transition blockade (Fig. 3C).

To examine the molecular mechanism of G1 arrest, we analyzed G1/S cell-cycle regulatory proteins by Western blotting. Apatinib dose-dependently reduced the phosphorylation of Rb and decreased expression of cyclins A1/A2, D1, and E1, as well as their partner cyclin-dependent kinases CDK2, CDK4, and CDK6 (Fig. 3D). In contrast, apatinib increased expression of the cyclin-dependent kinase inhibitor p27. However, E2F1 expression remained unchanged (Supplementary Fig. S1A), consistent with maintained Rb-mediated repression. We also observed reduced cyclin B1 expression, although its partner kinase CDK1 was unchanged (Fig. 3D). Together, these results demonstrate that apatinib suppresses SS cell proliferation by reducing G1/S phase regulators and inducing G1 phase arrest.

### Apatinib inhibits proliferation via the VEGFR2/AKT/FOXO3A pathway

**Fig. 4:**
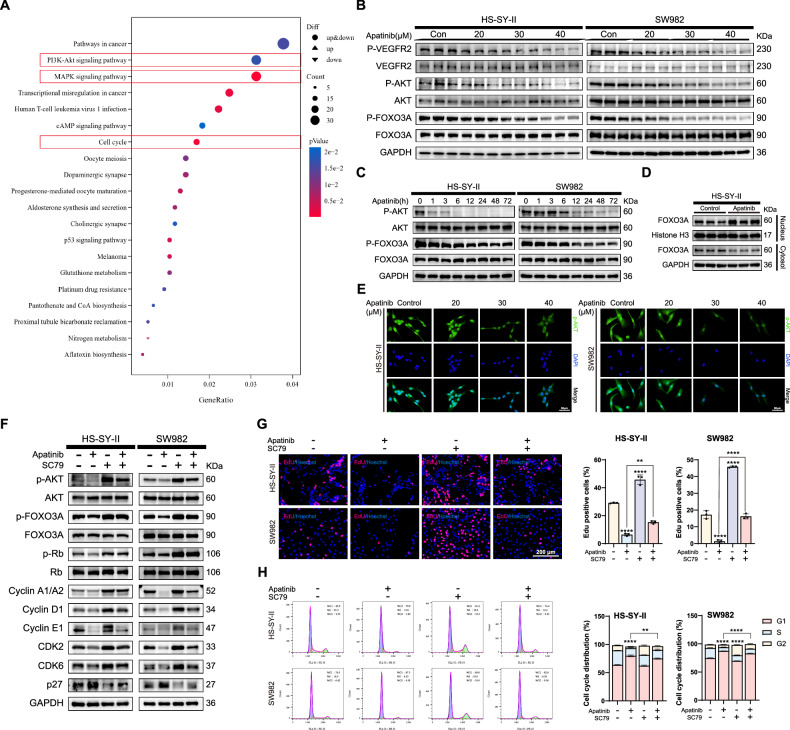
Apatinib inhibits proliferation through the VEGFR2/AKT/FOXO3A pathway. **A** KEGG pathway enrichment analysis of differentially expressed genes from transcriptome sequencing of HS-SY-II cells treated with apatinib (40 μM, 48 h). **B** Western blot analysis of p-VEGFR2 (Tyr1175), total VEGFR2, p-AKT (Ser473), total AKT, p-FOXO3A (Ser253), and total FOXO3A in HS-SY-II and SW982 cells treated with indicated concentrations of apatinib for 48 h. **C** Western blot analysis of p-AKT (Ser473), total AKT, p-FOXO3A (Ser253), and total FOXO3A in HS-SY-II and SW982 cells treated with apatinib (40 μM) for the indicated times. **D** Nuclear and cytoplasmic fractionation analysis of FOXO3A levels in HS-SY-II cells after apatinib treatment. Histone H3 and GAPDH serve as nuclear and cytoplasmic markers, respectively. **E** Representative immunofluorescence images of p-AKT (green) and DAPI (blue) in HS-SY-II and SW982 cells after apatinib treatment. Scale bar = 50 μm. **F** Western blot analysis of p-AKT, total AKT, p-FOXO3A, total FOXO3A, p-Rb, total Rb, cyclin A1/A2, cyclin D1, cyclin E1, CDK2, CDK6, and p27 in HS-SY-II and SW982 cells treated with apatinib (40 μM) with or without the AKT activator SC79 (8 μg/mL). **G** Representative images and quantification of EdU-positive cells in HS-SY-II and SW982 cells treated with apatinib (40 μM) with or without SC79 (8 μg/mL), assessed by EdU incorporation assay. Scale bar = 200 μm. **H** Representative flow cytometry plots and quantification of cell cycle distribution (G1, S, and G2 phases) in HS-SY-II and SW982 cells treated with apatinib (40 μM) with or without SC79 (8 μg/mL). Data are presented as mean ± SD (*n* = 3 biological replicates). Statistical significance was determined by one-way ANOVA. **p < 0.01, ****p < 0.0001.

To examine the mechanisms of G1 arrest, we performed transcriptome sequencing on apatinib-treated HS-SY-II cells. KEGG analysis showed that apatinib-altered genes were primarily enriched in the PI3K/AKT, MAPK, and cell-cycle pathways (Fig. 4A). VEGFR2 activates PI3K/AKT signaling, which regulates cell survival and cell-cycle progression [21]. AKT phosphorylates FOXO3A to promote its nuclear export, thereby preventing transcription of genes that block G1/S transition [22, 23]. Based on this VEGFR2-AKT-FOXO3A connection, we examined whether apatinib regulates this axis.

Western blot results showed that apatinib reduced VEGFR2 phosphorylation at Tyr1175 (Fig. 4B) and decreased the levels of p-AKT and p-FOXO3A in a time- and dose-dependent manner, while total protein levels remained unchanged (Fig. 4B, C). Because AKT activity regulates FOXO3A localization, we next analyzed FOXO3A subcellular distribution. Nuclear-cytoplasmic fractionation showed increased nuclear FOXO3A and decreased cytoplasmic FOXO3A after apatinib treatment, indicating FOXO3A reactivation (Fig. 4D). Immunofluorescence staining further confirmed the reduction of p-AKT signals (Fig. 4E).

To test whether AKT signaling mediates these effects, we performed rescue experiments using the AKT activator SC79. SC79 reversed the apatinib-induced reduction in p-AKT and p-FOXO3A and restored expression of G1/S regulators (p-Rb, cyclins A1/A2, D1, and E1, CDK2, and CDK6) while reducing p27 upregulation (Fig. 4F). SC79 also reversed the apatinib-induced decrease in HIF1-α expression (Supplementary Fig. S1B, C). Furthermore, SC79 partially restored EdU incorporation and reduced G1 phase accumulation (Fig. 4G, H). Together, these results demonstrate that apatinib inhibits SS cell-cycle progression by suppressing the VEGFR2/AKT/FOXO3A signaling axis.

### Apatinib inhibits proliferation and angiogenesis via the ERK1/2/FOXM1 pathway

**Fig. 5:**
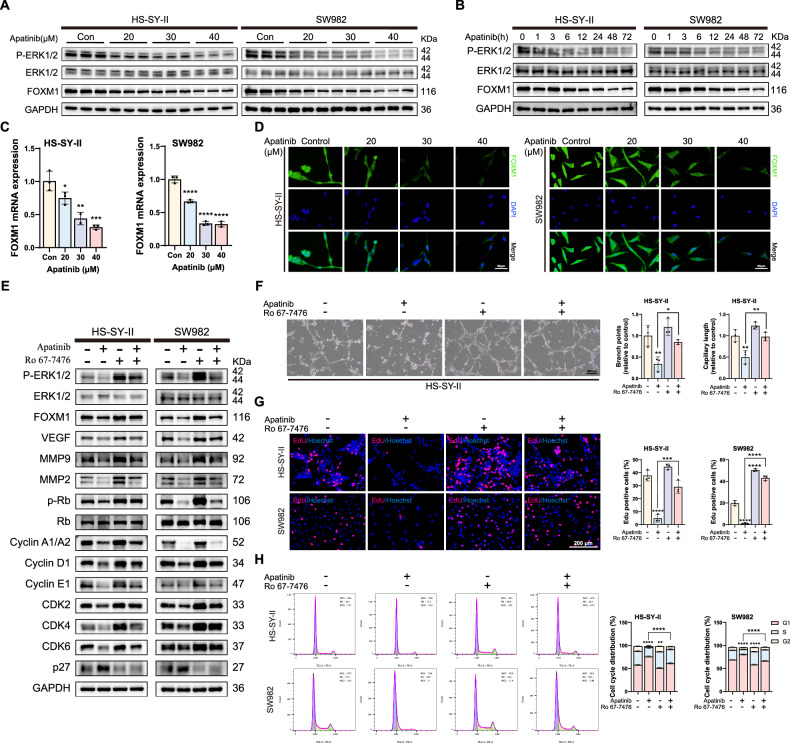
Apatinib suppresses angiogenic activity and G1/S progression via ERK1/2/FOXM1 signaling. **A** Western blot analysis of p-ERK1/2 (Thr202/Tyr204), total ERK1/2, and FOXM1 in HS-SY-II and SW982 cells treated with the indicated concentrations of apatinib for 48 h. **B** Western blot analysis of p-ERK1/2 (Thr202/Tyr204), total ERK1/2, and FOXM1 after apatinib treatment for the indicated times. **C** qPCR analysis of FOXM1 mRNA levels after apatinib treatment at indicated concentrations. **D** Representative immunofluorescence images of FOXM1 (green) and DAPI (blue) after apatinib treatment. Scale bar = 50 μm. **E** Western blot analysis of p-ERK1/2, total ERK1/2, FOXM1, VEGF, MMP9, MMP2, p-Rb, total Rb, cyclin A1/A2, cyclin D1, cyclin E1, CDK2, CDK4, CDK6, and p27 in HS-SY-II and SW982 cells treated with apatinib (40 μM) with or without the ERK1/2 activator Ro 67-7476 (1 μM). **F** Representative images and quantification of total tube length and branch points in HUVECs cultured with conditioned medium from HS-SY-II cells treated with apatinib (40 μM) with or without Ro 67-7476 (1 μM), assessed by tube formation assay. Scale bar = 200 μm. **G** Representative images and quantification of EdU-positive cells in HS-SY-II and SW982 cells treated with apatinib (40 μM) with or without Ro 67-7476 (1 μM), assessed by EdU incorporation assay. Scale bar = 200 μm. **H** Representative flow cytometry plots and quantification of cell cycle distribution (G1, S, and G2 phases) in HS-SY-II and SW982 cells treated with apatinib (40 μM) with or without Ro 67-7476 (1 μM). Data are presented as mean ± SD (*n* = 3 biological replicates). Statistical significance was determined by one-way ANOVA. *p < 0.05, **p < 0.01, ***p < 0.001, ****p < 0.0001.

Since apatinib inhibits both cell growth and angiogenesis, we investigated whether these processes share common molecular pathways. KEGG analysis of our transcriptome data showed that the MAPK pathway was another major pathway altered following apatinib treatment (Fig. 4A). ERK1/2, a core component of the MAPK pathway [27], promotes the expression and activity of FOXM1, a transcription factor that regulates cell cycle progression [25]. FOXM1 also drives expression of angiogenesis-related genes such as VEGF in multiple tumor types [25, 26]. We therefore examined whether apatinib affects the ERK1/2/FOXM1 axis. Western blotting showed that apatinib reduced p-ERK1/2 in a concentration-dependent manner, while total ERK1/2 levels were largely unchanged. FOXM1 was also decreased at both mRNA and protein levels (Fig. 5A, C). Similar changes were observed over time, with reduced p-ERK1/2 and downregulated FOXM1 after apatinib treatment at multiple time points (Fig. 5B). Immunofluorescence staining confirmed reduced FOXM1 signals in apatinib-treated cells (Fig. 5D).

To test whether ERK1/2 activity was required for these downstream changes, we used the ERK1/2 activator Ro 67-7476. Ro 67-7476 partially restored p-ERK1/2 and FOXM1 levels, reversed apatinib-induced reductions in angiogenesis-related factors (VEGF, MMP9, MMP2) and cell-cycle regulators (p-Rb, G1/S regulators), and reduced p27 upregulation (Fig. 5E). Ro 67-7476 also partially rescued tube formation, increased EdU incorporation, and alleviated G1 phase accumulation (Fig. 5F–H). Together, these results demonstrate that apatinib suppresses both cell-cycle progression and angiogenesis by inhibiting the ERK1/2/FOXM1 signaling axis.

### Apatinib inhibits angiogenesis by suppressing the VEGFR2/AKT/eNOS/NO pathway in endothelial cells

**Fig. 6:**
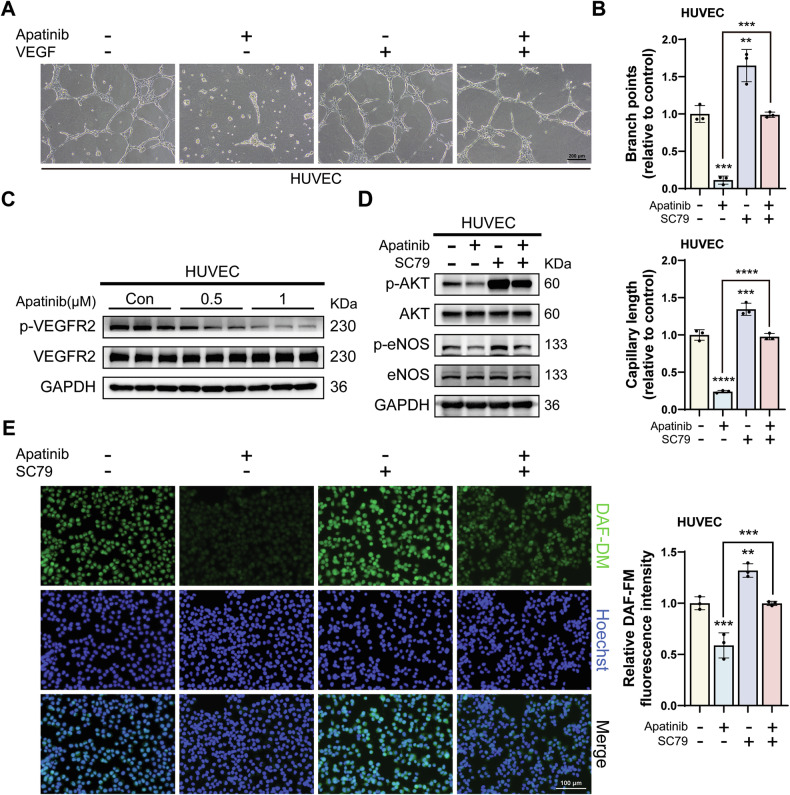
Apatinib directly inhibits endothelial tube formation through the VEGFR2/AKT/eNOS/NO pathway. **A**, **B** Representative images and quantification of tube length and branch points in HUVECs treated with apatinib (1 μM) with or without VEGF (100 ng/mL), assessed by tube formation assay. Scale bar = 200 μm. **C** Western blot analysis of p-VEGFR2 (Tyr1175) and total VEGFR2 in HUVECs treated with the indicated concentrations of apatinib. **D** Western blot analysis of p-AKT (Ser473), total AKT, p-eNOS (Ser1177), and total eNOS in HUVECs treated with apatinib (1 μM) with or without the AKT activator SC79 (8 μg/mL). **E** Representative fluorescence images and quantification of DAF-FM DA fluorescence intensity reflecting intracellular NO levels in HUVECs treated with apatinib (1 μM) with or without SC79 (8 μg/mL). Scale bar = 100 μm. Data are presented as mean ± SD (*n* = 3 biological replicates). Statistical significance was determined by one-way ANOVA. **p < 0.01, ***p < 0.001, ****p < 0.0001.

To examine the direct anti-angiogenic effect of apatinib on endothelial cells, we performed tube formation assays using HUVECs. VEGF stimulation served as a positive control and increased tube-like structures, whereas 1 μM of apatinib markedly reduced tube formation (Fig. 6A, B), indicating that apatinib directly suppresses endothelial angiogenic activity.

VEGF drives angiogenesis primarily through VEGFR2 [10]. Activation of VEGFR2 engages PI3K/AKT signaling, which promotes eNOS phosphorylation and NO production [10, 13]. This VEGFR2/AKT/eNOS/NO cascade supports key endothelial migration and tube formation [13]. We therefore examined whether apatinib affects this signaling pathway. Western blot analysis showed that apatinib reduced p-VEGFR2 levels in a concentration-dependent manner, while total VEGFR2 remained unchanged (Fig. 6C). Apatinib also decreased p-AKT and p-eNOS levels (Fig. 6D).

To test whether AKT signaling mediates these effects, we used the AKT activator SC79 for rescue experiments. SC79 restored p-AKT and p-eNOS levels in apatinib-treated HUVECs (Fig. 6D). DAF-FM DA staining showed that apatinib reduced intracellular NO, but SC79 partially restored NO levels (Fig. 6E). Together, these results indicate that apatinib inhibits endothelial tube formation by suppressing the VEGFR2/AKT/eNOS/NO signaling pathway.

### Apatinib suppresses SS tumor growth in vivo

**Fig. 7:**
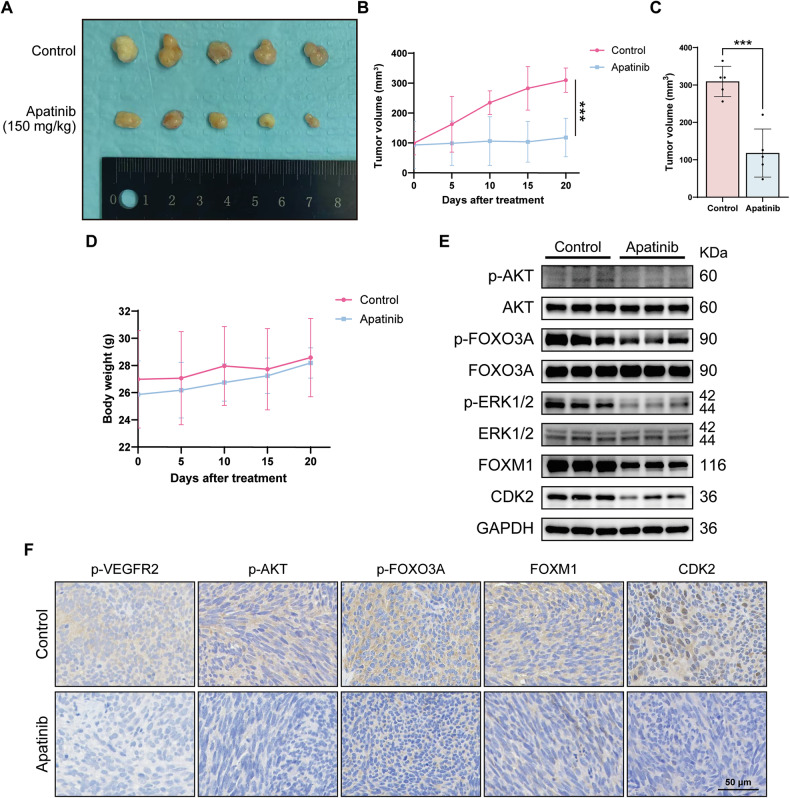
Apatinib inhibits SS growth in vivo and suppresses AKT/FOXO3A and ERK1/2/FOXM1 signaling. **A** Representative image of xenograft tumors from mice treated with vehicle or apatinib (150 mg/kg daily, oral gavage). **B** Tumor growth curves showing tumor volume measured every 5 days during the 20-day treatment period. **C** Quantification of tumor volume at the endpoint of the treatment period. **D** Body weight of mice recorded during the treatment period. **E** Western blot analysis of p-AKT (Ser473), total AKT, p-FOXO3A (Ser253), total FOXO3A, p-ERK1/2 (Thr202/Tyr204), total ERK1/2, FOXM1, and CDK2 in tumor lysates from vehicle- and apatinib-treated mice. **F** Representative immunohistochemical images and quantification of p-VEGFR2 (Tyr1175), p-AKT (Ser473), p-FOXO3A (Ser253), FOXM1, and CDK2 staining intensity in xenograft tumors from vehicle- and apatinib-treated mice. Scale bar = 50 μm. Data are presented as mean ± SD (n = 5 biological replicates). Statistical significance was determined by the Student’s t-test. ***p < 0.001.

To evaluate the antitumor effect of apatinib in vivo, we established a xenograft model by subcutaneously injecting HS-SY-II cells into BALB/c nude mice. Once tumors were established, mice were randomized to receive either vehicle or 150 mg/kg apatinib daily via oral gavage. After 20 days of treatment, apatinib markedly suppressed tumor growth, and tumor volume was significantly reduced compared with the control group (Fig. 7A–C). Furthermore, no obvious changes in body weight were observed between the two groups, suggesting that the treatment was well tolerated (Fig. 7D).

We next examined whether the signaling changes observed in vitro occurred in tumor tissues. Western blot analysis of xenograft samples showed that apatinib reduced the levels of p-AKT, p-FOXO3A, p-ERK1/2, FOXM1, and CDK2 (Fig. 7E). Consistent with these findings, immunohistochemistry confirmed a reduction in the staining intensity of p-VEGFR2, p-AKT, p-FOXO3A, FOXM1, and CDK2 in the apatinib-treated group (Fig. 7F). Together, these results demonstrate that apatinib suppresses SS growth in vivo by targeting the VEGFR2/AKT/FOXO3A and ERK1/2/FOXM1 signaling pathways.

## Discussion

**Fig. 8:**
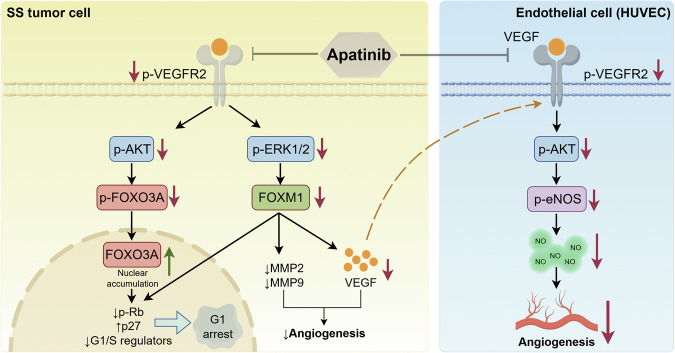
Schematic illustration of apatinib-mediated inhibition of SS growth through dual VEGFR2-dependent signaling pathways. Apatinib inhibits VEGFR2 phosphorylation, suppressing two parallel pathways: (1) AKT/FOXO3A, which promotes FOXO3A nuclear translocation and G1/S arrest; and (2) ERK1/2/FOXM1, which suppresses transcription of cell cycle regulators and angiogenic factors (VEGF, MMP2, MMP9). Additionally, apatinib inhibits the VEGFR2/AKT/eNOS/NO axis in endothelial cells, impairing angiogenesis. These coordinated effects suppress SS growth in vivo.

SS remains a clinically challenging soft-tissue malignancy with high rates of local recurrence and metastatic spread, and outcomes are poor once disease becomes unresectable or metastatic [2–4]. Current systemic therapies provide limited objective responses, and resistance is common [4]. Anti-angiogenic therapy represents a rational approach, as elevated VEGF signaling is associated with adverse outcomes in SS [11, 12]. We therefore investigated apatinib, a VEGFR2-directed TKI with clinical activity in multiple solid tumors and emerging data in advanced sarcoma [19]. Here, we demonstrate that apatinib suppresses SS proliferation and metastatic behavior while blocking angiogenesis. Mechanistically, apatinib inhibits the VEGFR2/AKT/FOXO3A and ERK1/2/FOXM1 pathways, causing G1 phase arrest and reduced angiogenesis in SS cells. Apatinib also directly blocks endothelial angiogenic function via the VEGFR2/AKT/eNOS/NO pathway (Fig. 8). These findings reveal a dual mechanism in which apatinib controls tumor-cell proliferation and endothelial angiogenesis, supporting VEGFR2 inhibition as a therapeutic strategy for SS.

In this study, we found that apatinib reduced viability and induced programmed cell death in SS cells. Apatinib increased cleaved caspases and Bax levels while reducing Bcl-2, confirming apoptosis. Apatinib also triggered autophagy, as evidenced by increased LC3 processing and ATG5 expression coupled with p62 degradation. This dual activation of apoptosis and autophagy aligns with findings in osteosarcoma [28], colorectal [29] and lung cancers [30]. Importantly, autophagy can be cytotoxic or cytoprotective. Accumulating evidence suggests that apatinib-induced autophagy serves a cytoprotective role, as its pharmacological inhibition sensitizes cells to apoptosis [28, 29]. Therefore, determining whether autophagy facilitates apatinib-mediated cytotoxicity or serves as an adaptive resistance mechanism warrants further investigation.

In addition to inducing programmed cell death, apatinib reduced SS cell migration and invasion in wound-healing and Transwell assays. Apatinib reversed EMT by increasing E-cadherin while decreasing N-cadherin, Vimentin, Snail, ZEB1, and MMP2/MMP9. These findings support that VEGF/VEGFR signaling promotes malignant phenotypes through non-angiogenic mechanisms, including enhanced migration and invasion [21]. Apatinib also reduced VEGF expression in SS cells and inhibited endothelial tube formation, linking these tumor-cell alterations to impaired angiogenesis. Similar effects have been reported in hepatocellular carcinoma [31], thyroid cancer [32], and cholangiocarcinoma [33]. These data suggest that apatinib restrains SS progression by suppressing EMT and angiogenesis, two processes that cooperate to drive tumor spread and metastasis [34].

We further investigated the anti-proliferative effects of apatinib in SS cells. Apatinib markedly reduced clonogenic growth and DNA synthesis, as evidenced by fewer colonies and decreased EdU incorporation. Cell-cycle analysis revealed clear G1 accumulation and S-phase depletion, indicating G1/S blockade. Molecularly, these changes were accompanied by reduced p-Rb and coordinated downregulation of core G1/S regulators, together with upregulation of the CDK inhibitor p27. These changes are consistent with the standard regulation of the G1 phase, in which cyclin D/E-dependent CDKs drive progressive Rb phosphorylation to enable E2F-dependent S-phase entry, whereas CKIs such as p27 antagonize this process [35]. Our observation that apatinib enforces G1 arrest aligns with prior reports in lung cancer and osteosarcoma [28, 30]. We also observed reduced cyclin B1 with largely unchanged CDK1, a pattern expected from a primary G1/S blockade that limits progression into G2/M and thereby lowers mitotic cyclin B1 abundance [36, 37].

The VEGFR2/AKT/FOXO3A axis directly accounts for the G1 arrest observed. Canonically, AKT phosphorylates FOXO transcription factors, promoting their inactivation via nuclear exclusion and 14-3-3 binding [38]. FOXO3A enforces the G1/S checkpoint by upregulating CKIs (p21 and p27) and repressing cyclin D1, thereby inhibiting Cyclin D-CDK4/6 and Cyclin E/A-CDK2, maintaining hypo-phosphorylated Rb and blocking E2F-driven S-phase transcription [23, 35, 39]. In our models, apatinib decreased p-AKT and p-FOXO3A, increased nuclear FOXO3A accumulation, and upregulated p27 while broadly downregulated G1/S regulators and reduced p-Rb. SC79 rescue partially restored p-AKT/p-FOXO3A and reversed these cell-cycle changes, confirming that AKT/FOXO3A inhibition causally drives apatinib-induced G1 blockade. Apatinib also reduced HIF1-α expression in an AKT-dependent manner. As a key AKT effector, HIF1-α promotes angiogenesis by regulating pro-angiogenic factors including VEGF [40, 41], linking the AKT pathway to angiogenic activity in SS. Although PI3K/AKT signaling is known to be essential for SS progression [42] and FOXO3A contributes to HDAC-inhibitor-induced arrest and cell death in SS models [43], direct mechanistic connections between FOXO3A and cell-cycle control in SS have remained limited. By positioning FOXO3A as a critical downstream effector of VEGFR2/AKT inhibition, our findings establish a clear molecular route from receptor blockade to G1 phase arrest in SS.

A second major finding is suppression of the ERK1/2/FOXM1 axis, which mechanistically links reduced proliferation to downregulation of angiogenesis-related genes. ERK signaling activates FOXM1 by enhancing its transcriptional activity and sustaining its expression [25]. FOXM1 is a well-established cell-cycle transcription factor that also drives angiogenic programs, including direct upregulation of VEGF [44–46]. FOXM1 is overexpressed across multiple cancers and sarcomas [47] and functions as an independent prognostic factor in SS, positioning its inhibition as a promising therapeutic strategy [47]. Mechanistic studies on FOXM1 blockade in SS, however, remain limited. In our study, apatinib reduced p-ERK1/2 and FOXM1 expression at both mRNA and protein levels, and ERK1/2 reactivation partially restored the expression of FOXM1, VEGF, MMP2, MMP9 and multiple G1/S regulatory factors, as well as tube formation capacity and proliferative readouts. Together, these data identify the ERK1/2/FOXM1 pathway as a central node through which apatinib coordinately suppresses tumor proliferation and angiogenic signaling in SS.

A key advance of our study is the finding that apatinib simultaneously suppresses the two principal VEGFR2 effector pathways: PI3K/AKT and MAPK/ERK. These pathways are not independent; they exhibit extensive crosstalk in which inhibition of one frequently triggers compensatory activation of the other, a well-documented driver of acquired resistance [48–50]. Dual pathway blockade by apatinib therefore offers the potential for more durable clinical responses. At the transcription level, FOXO3A and FOXM1 function as an antagonistic switch: FOXO3A enforces cell-cycle restraint, whereas FOXM1 promotes proliferation and angiogenic gene programs [23, 44]. FOXO3A activation is known to repress FOXM1 expression and activity and to directly antagonize FOXM1 at shared target promoters, including VEGF [51, 52]. Our data show that apatinib concurrently activated FOXO3A and suppressed FOXM1. This dual regulation is not additive but mutually reinforcing: FOXO3A further represses the FOXM1/VEGF axis, stabilizing G1 phase arrest and reduced angiogenic output. The resulting synergy substantially impedes compensatory reactivation of tumor survival circuits.

Our endothelial-cell experiments further clarify the direct anti-angiogenic mechanism of apatinib. VEGF signaling through VEGFR2 activates PI3K/AKT, promoting eNOS phosphorylation and NO production that drive endothelial migration and tube formation [13, 14]. Consistent with this cascade, apatinib blocked VEGF-induced responses in HUVECs by suppressing phosphorylation of VEGFR2, AKT, and eNOS, leading to decreased NO levels and impaired tube formation. AKT reactivation partially restored p-eNOS and NO production, confirming the VEGFR2/AKT/eNOS/NO axis as a primary target of apatinib’s anti-angiogenic action. The VEGFR2/AKT/eNOS/NO axis is also essential for endothelial homeostasis and vasodilation. Its inhibition is implicated in apatinib-related vascular adverse events, particularly treatment-emergent hypertension [53]. VEGF/VEGFR inhibitors are known to induce hypertension by impairing endothelial NO signaling while increasing vasoconstrictor tone, thereby increasing peripheral resistance [53]. In line with this, a preclinical study of apatinib reported elevated blood pressure together with reduced eNOS, linking endothelial dysfunction and reduced NO bioavailability to apatinib-associated hypertension [54]. Although these findings derive from in vitro HUVEC models, clinical vascular toxicities involve systemic and multi-organ factors and therefore require in vivo validation. Nonetheless, our findings identify the VEGFR2/AKT/eNOS/NO axis as a defined endothelial target of apatinib, providing mechanistic insight into its anti-angiogenic efficacy and a foundation for understanding associated vascular toxicities.

Although our study elucidates the mechanisms by which apatinib suppresses SS growth and angiogenesis, several limitations should be acknowledged. First, these effects were demonstrated in SS cell lines and xenograft models; validation in larger, more heterogeneous patient-derived cohorts will be required to establish clinical relevance. Second, although pharmacological activators were used to implicate the VEGFR2/AKT/FOXO3A and ERK1/2/FOXM1 pathways, apatinib is a multi-kinase inhibitor and potential off-target effects cannot be excluded. Future genetic approaches will be essential to confirm causality. Finally, while apatinib exhibited potent antitumor and anti-angiogenic activity in SS, its efficacy and underlying mechanisms in other sarcoma subtypes and solid tumors remain to be clarified. Expanding these studies to a broader range of tumor models and clinical samples will clarify the generalizability of our findings and help identify the patient populations most likely to benefit from VEGFR2-targeted therapy.

In summary, our study demonstrates that apatinib exerts dual antitumor effects in SS. It simultaneously suppresses tumor-cell proliferation and angiogenic signaling by inhibiting the VEGFR2-mediated AKT/FOXO3A and ERK1/2/FOXM1 axes, while directly impairing endothelial angiogenic function via the VEGFR2/AKT/eNOS/NO pathway. These findings identify apatinib as a promising therapeutic agent for SS.

## Materials and methods

### Cell lines and cell culture

Human synovial sarcoma (SS) cell lines (HS-SY-II and SW982) were kindly provided by Professor Wei Guo from Peking University People’s Hospital. Human umbilical vein endothelial cells (HUVECs) were purchased from iCell Bioscience Inc. (Shanghai, China). HS-SY-II and SW982 cells were cultured in DMEM (MeilunBio, Liaoning, China), and HUVECs in RPMI-1640 (BasalMedia, Shanghai, China), both supplemented with 10% fetal bovine serum (FBS, NCM, Suzhou, China), 100 IU/mL penicillin G, and 100 μg/mL streptomycin at 37 °C in a humidified incubator with 5% CO₂.

Cells were treated with indicated concentrations of apatinib (0, 20, 30, 40 μM), with 0.1% dimethyl sulfoxide (DMSO) serving as vehicle control. For rescue experiments, cells were co-treated with apatinib and either 8 μg/mL of SC79 (MCE, Shanghai, China) or 1 μM of Ro 67-7476 (MCE, Shanghai, China). Apatinib (Hengrui Medicine Co. Ltd., Jiangsu, China) was dissolved in DMSO (Biosharp, Beijing, China).

### Cell viability assay

Cell viability was assessed using the Cell Counting Kit-8 (CCK-8) assay (GLPBIO, Montclair, USA). Briefly, HS-SY-II and SW982 cells (1 × 10^4^ cells/well) were cultured in 96-well plates and treated with apatinib at indicated concentrations (0, 2, 5, 10, 20, 30, 40, 80 μM) for 24, 48, or 72 h. CCK-8 reagent was then added, and cells were incubated for an additional 3 h at 37 °C. Absorbance was measured at 450 nm using a microtiter reader, and values were normalized to vehicle-treated controls.

### Calcein-AM/PI staining

Cells were treated with apatinib for 48 h and stained using a Calcein-AM/PI Live/Dead Viability Assay Kit (Beyotime, Shanghai, China) according to the manufacturer’s instructions. Cells were incubated with Calcein-AM/PI working solution for 30 min in the dark, and fluorescence images were immediately captured using a fluorescence microscope (Olympus, Tokyo, Japan). The percentage of PI-positive cells was quantified using ImageJ software (NIH, USA).

### Flow cytometry analysis of apoptosis

Apoptosis was analyzed using an Annexin V-FITC/PI apoptosis detection kit (Beyotime). Following apatinib treatment for 48 h, cells were collected, washed with PBS, resuspended in binding buffer, and stained with Annexin V-FITC and PI for 10 min in the dark. Apoptotic cells were analyzed using a CytoFLEX LX (Beckman Coulter, Brea, USA), and data were analyzed using CytExpert 2.4.0 software.

### Colony formation assay

Cells (5 × 10^3^ cells/well) were seeded in 6-well plates, treated with apatinib for 48 h, and cultured for an additional 14 days. Colonies were washed with PBS, fixed with 4% paraformaldehyde, and stained with 0.1% crystal violet (15 min each). Colonies containing >50 cells were counted using ImageJ software.

### EdU incorporation assay

DNA synthesis was evaluated using an EdU Cell Proliferation Kit with AF555 (Beyotime). Cells (1 × 10^3^ cells/well) were plated in 96-well plates, treated with apatinib for 48 h with or without rescue reagents, and incubated with EdU (10 μM) for 2 h. Cells were then fixed with 4% paraformaldehyde for 15 min, permeabilized with 0.3% Triton X-100 for 10 min, and EdU incorporation was detected via click chemistry according to the manufacturer’s protocol. Nuclei were counterstained with Hoechst 33342. Fluorescence images were captured using a fluorescence microscope (Olympus), and EdU-positive cells were assessed using ImageJ software.

### Cell cycle analysis

Cell cycle distribution was measured using a Cell Cycle Analysis Kit (Beyotime). Following apatinib treatment for 48 h with or without rescue reagents, cells were collected, washed with PBS, and fixed in pre-cooled 70% ethanol at 4 °C for 30 min. Cells were then washed, treated with RNase A and PI for 30 min in the dark, and analyzed using a CytoFLEX LX (Beckman Coulter). Cell cycle distribution was quantified using FlowJo 10.8.1 software.

### Wound healing assay

Cells were seeded in 6-well plates and grown to 90–100% confluence. Monolayers were scratched with a sterile 200 μL pipette tip to generate a linear wound. After washing with PBS to remove cellular debris, cells were cultured in 3% FBS-DMEM containing apatinib at concentrations of 0, 20, 30, and 40 μM. Images were captured at 0 h and 48 h using an inverted microscope, and the wound area was measured and quantified using ImageJ software.

### Transwell assay

Following treatment with apatinib (0, 20, 30, 40 μM) for 48 h, cells were resuspended to 1 × 10^5^ cells/well and seeded into the upper chambers of 24-well Transwell plates (8 μm pore; Corning, New York, USA) in serum-free medium. For invasion assays, the upper chambers were precoated with Matrigel (Corning). Migration assays were performed without Matrigel coating. The lower chambers were filled with 800 μL of medium containing 30% FBS as a chemoattractant. After 24 h, non-migrated cells were removed from the upper surface with cotton swabs, and cells on the lower membrane surface were fixed with 4% paraformaldehyde and stained with 0.1% crystal violet. Migrated and invaded cells were imaged by microscopy and quantified using ImageJ software.

### Tube formation assay

For the indirect angiogenesis assay, conditioned medium (CM) was collected from HS-SY-II cells grown in serum-free medium treated with or without apatinib for 48 h and centrifuged to remove cellular debris. HUVECs were then cultured in CM and seeded onto 12-well plates precoated with Matrigel (20 μL/well; Corning) at 2 × 10^5^ cells/well. For the direct endothelial treatment assay, HUVECs were pretreated with apatinib (1 μM) for 24 h before seeding onto Matrigel-coated plates. Recombinant human VEGF-A (100 ng/mL; MCE, Shanghai, China) was used as a positive control where indicated. After 24 h of incubation at 37 °C with 5% CO₂, tube formation was imaged by microscope and quantified using ImageJ software.

### NO detection

Intracellular NO production was assessed using a Nitric Oxide Assay Kit with DAF-FM DA (Beyotime). HUVECs were treated with apatinib (1 μM) alone or in combination with the AKT activator SC79 (8 μg/mL) for 24 h, washed with PBS, and incubated with DAF-FM DA at 37 °C for 20 min in the dark. Nuclei were counterstained with Hoechst 33342 (5 μg/mL) for 10 min. Cells were then washed with PBS and immediately imaged using a fluorescence microscope (Olympus). Fluorescence intensity was quantified using ImageJ software.

### RNA sequencing and pathway enrichment analysis

HS-SY-II cells were treated with 40 μM apatinib or vehicle (0.1% DMSO) for 48 h. Total RNA was extracted using TRIzol (Invitrogen, Carlsbad, USA) according to the manufacturer’s protocol. RNA quality and quantity were assessed, followed by library construction and RNA sequencing performed by Tsingke Biological Technology (Beijing, China). Differentially expressed genes (DEGs) were identified using adjusted *P* < 0.05 and |fold change|> 2 as thresholds. Gene Ontology (GO) and Kyoto Encyclopedia of Genes and Genomes (KEGG) pathway enrichment analyses were performed to identify significantly enriched biological processes and signaling pathways.

### Nuclear-cytoplasmic fractionation

To assess FOXO3A subcellular localization, nuclear and cytoplasmic proteins were separated using a Nuclear and Cytoplasmic Protein Extraction Kit (Beyotime) according to the manufacturer’s protocol. Following treatment, cells were harvested, washed with ice-cold PBS, and fractionated on ice. Protein concentrations in both fractions were quantified using a BCA Protein Assay Kit (Glpbio, Montclair, USA), and equal amounts of protein were analyzed by western blot. Histone H3 and GAPDH served as nuclear and cytoplasmic markers, respectively. The antibodies used are listed in Supplementary Table S1.

### Quantitative real-time PCR (qRT-PCR)

Total RNA was isolated using an RNA Extraction Kit (Gooniebio, Guangzhou, China) according to the manufacturer’s protocol. First-strand cDNA was synthesized from 1 μg of total RNA using a cDNA Synthesis Kit (Gooniebio). qRT-PCR was performed using 2× qPCR Master Mix (Gooniebio) on a real-time PCR system (Thermo Fisher Scientific, Waltham, USA). The cycling conditions were as follows: 95 °C for 10 min, followed by 40 cycles of 95 °C for 15 s and 60 °C for 1 min. All reactions were performed in triplicate. GAPDH served as an internal control, and relative expression levels were calculated by the 2^−ΔΔCt^ method. The primer sequences were as follows: FOXM1 forward: 5′-CGTCGGCCACTGATTCTCAAA-3′; reverse: 5′-GGCAGGGGATCTCTTAGGTTC-3′. GAPDH forward: 5′-GGAGCGAGATCCCTCCAAAAT-3′; reverse: 5′-GGCTGTTGTCATACTTCTCATGG-3′.

### Western blot analysis

Total proteins were extracted from cells and tumor tissues using RIPA Lysis Buffer (Epizyme, Shanghai, China) supplemented with protease and phosphatase inhibitors (Epizyme) on ice. Protein concentrations were determined using a BCA Protein Assay Kit. Equal amounts of protein (20 μg per lane) were separated by SDS-PAGE and transferred onto PVDF membranes (Vazyme, Nanjing, China). Membranes were blocked with 5% non-fat milk in TBST for 1 h at room temperature and then incubated with primary antibodies overnight at 4 °C. After washing three times with TBST, membranes were incubated with HRP-conjugated secondary antibodies (1:5000) for 1 h at room temperature. Protein bands were visualized using an enhanced chemiluminescence (ECL) kit (Biosharp, Beijing, China) and quantified by densitometry using ImageJ. GAPDH served as a loading control. The antibodies used are listed in Supplementary Table S1.

### Immunofluorescence (IF) staining

Cells on coverslips were treated with apatinib for 48 h, fixed with 4% paraformaldehyde for 15 min, permeabilized with 0.3% Triton X-100 for 10 min, and blocked with 10% goat serum for 1 h. Cells were incubated with primary antibodies overnight at 4 °C, followed by fluorescence-conjugated secondary antibodies (1:500) for 1 h in the dark. Nuclei were stained with DAPI (5 μg/mL) for 5 min. Images were captured using a fluorescence microscope (Olympus), and fluorescence intensity was quantified using ImageJ. Antibodies are listed in Supplementary Table S1.

### Tumor xenograft model

BALB/c nude mice (SPF grade, 4–5 weeks old, male) were purchased from GemPharmatech Co., Ltd. (Nanjing, China). A total of 5 × 10⁶ HS-SY-II cells in 100 μL PBS were subcutaneously injected into the right axilla of each mouse. Twenty-one days post-injection, when tumors reached a palpable size (~100 mm³), mice were randomly assigned to the control or the apatinib treatment group (*n* = 5 for each group). Apatinib (150 mg/kg/day) was dissolved in DMSO (stock), diluted in saline to achieve a final DMSO concentration of 2%, and administered daily by oral gavage for 20 consecutive days; the control group received vehicle solution (2% DMSO in saline) under identical conditions. Tumor dimensions (length and width) and body weight were measured every 5 days, and tumor volume was calculated using the formula: (length × width²)/2. On day 21 of treatment, mice were sacrificed and tumors were dissected, weighed, and processed for western blot analysis or histological evaluation.

### Immunohistochemistry (IHC)

Paraffin-embedded tumor tissue sections (4 μm) were deparaffinized, rehydrated, and subjected to heat-mediated antigen retrieval in citrate buffer (pH 6.0, 95 °C, 20 min). Endogenous peroxidase was blocked with 3% H₂O₂ for 10 min. After blocking with 10% goat serum for 1 h, sections were incubated with primary antibodies overnight at 4 °C, followed by HRP-conjugated secondary antibodies for 1 h at room temperature. Signals were visualized using DAB and counterstained with hematoxylin. Images were captured under an optical microscope, and antibody information is provided in Supplementary Table S1.

### Statistical analysis

Data were presented as mean ± standard deviation (SD) from at least three independent experiments. Comparisons between two groups were performed by unpaired two-tailed Student’s *t* test, and multiple-group comparisons were analyzed by one-way ANOVA followed by Tukey’s post-hoc test. *P* < 0.05 was considered statistically significant. Statistical analyses were conducted using GraphPad Prism (version 10).

## Supplementary information

Supplementary Figure

Supplementary Table

Original Western blot file

## Data Availability

The bulk RNA-seq data generated in this study have been deposited in the GSA-human database (https://ngdc.cncb.ac.cn/gsa-human/) under accession number HRA016952. Processed data and bioinformatic analysis codes are available from the corresponding author upon reasonable request.
