# Supplementary material for: Apatinib inhibits synovial sarcoma progression and angiogenesis via VEGFR2-mediated AKT/FOXO3A and ERK1/2/FOXM1 signaling pathways
Source: Cell Death Discov. 2026 Jun 3;12:328. doi: 10.1038/s41420-026-03188-7 (PMC13442848; doi:10.1038/s41420-026-03188-7)
Supplement: Supplementary file 1 [file 41420_2026_3188_MOESM1_ESM.pdf]

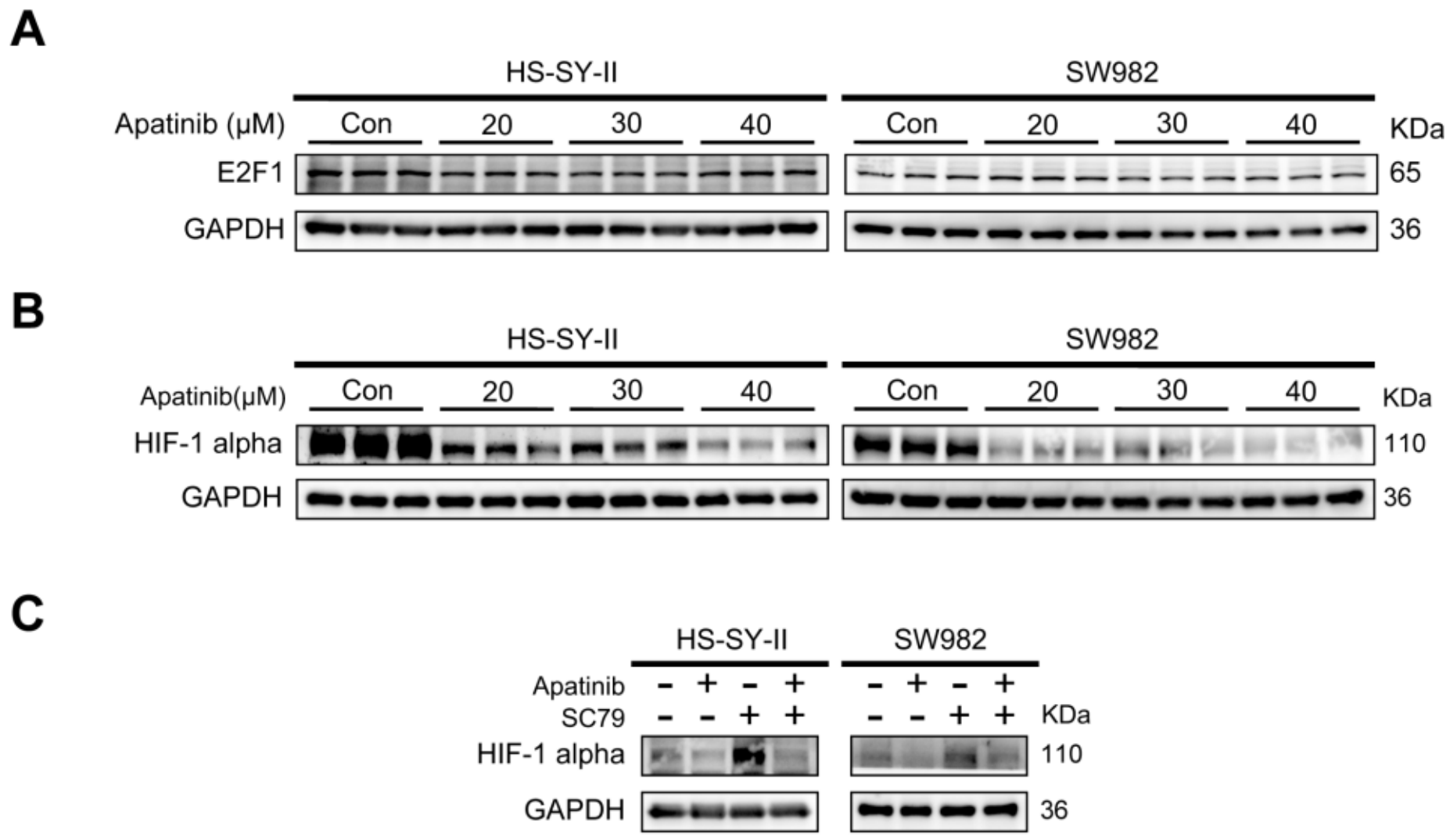

Figure S1 Effects of apatinib on E2F1 and HIF-1α expression. **A** Western blot analysis showing that apatinib does not alter E2F1 expression levels in SS cells. **B** Western blot analysis showing that dose-dependent reduction of HIF-1α expression in SS cells treated with apatinib for 48 h. **C** Western blot analysis showing that the AKT activator SC79 partially reverses apatinib-induced HIF-1α downregulation, indicating that AKT signaling mediates apatinib's inhibitory effect on HIF-1α expression. GAPDH serves as loading control. Data represent results from three independent experiments.
