# Supplementary material for: Apatinib inhibits synovial sarcoma progression and angiogenesis via VEGFR2-mediated AKT/FOXO3A and ERK1/2/FOXM1 signaling pathways
Source: Cell Death Discov. 2026 Jun 3;12:328. doi: 10.1038/s41420-026-03188-7 (PMC13442848; doi:10.1038/s41420-026-03188-7)

Figure 1D

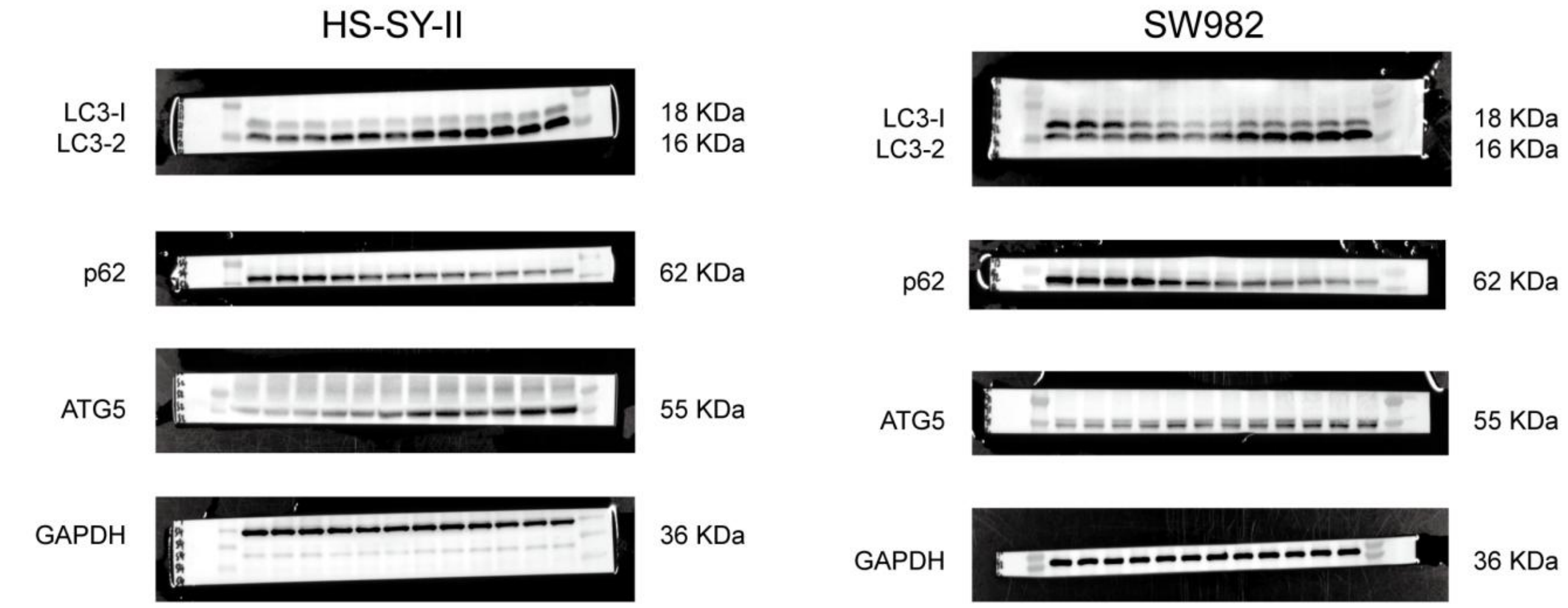

Figure 1E

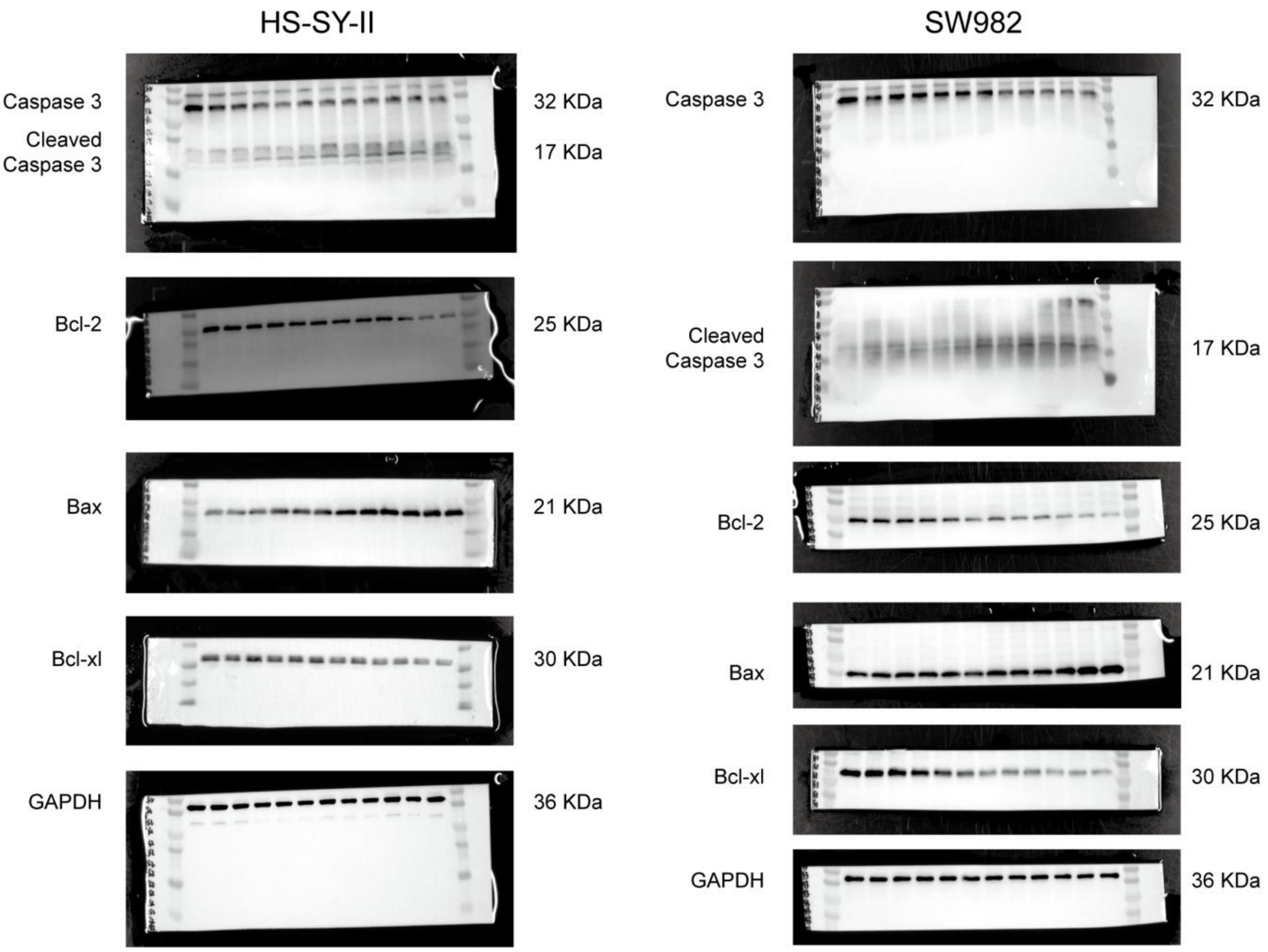

Figure 2G

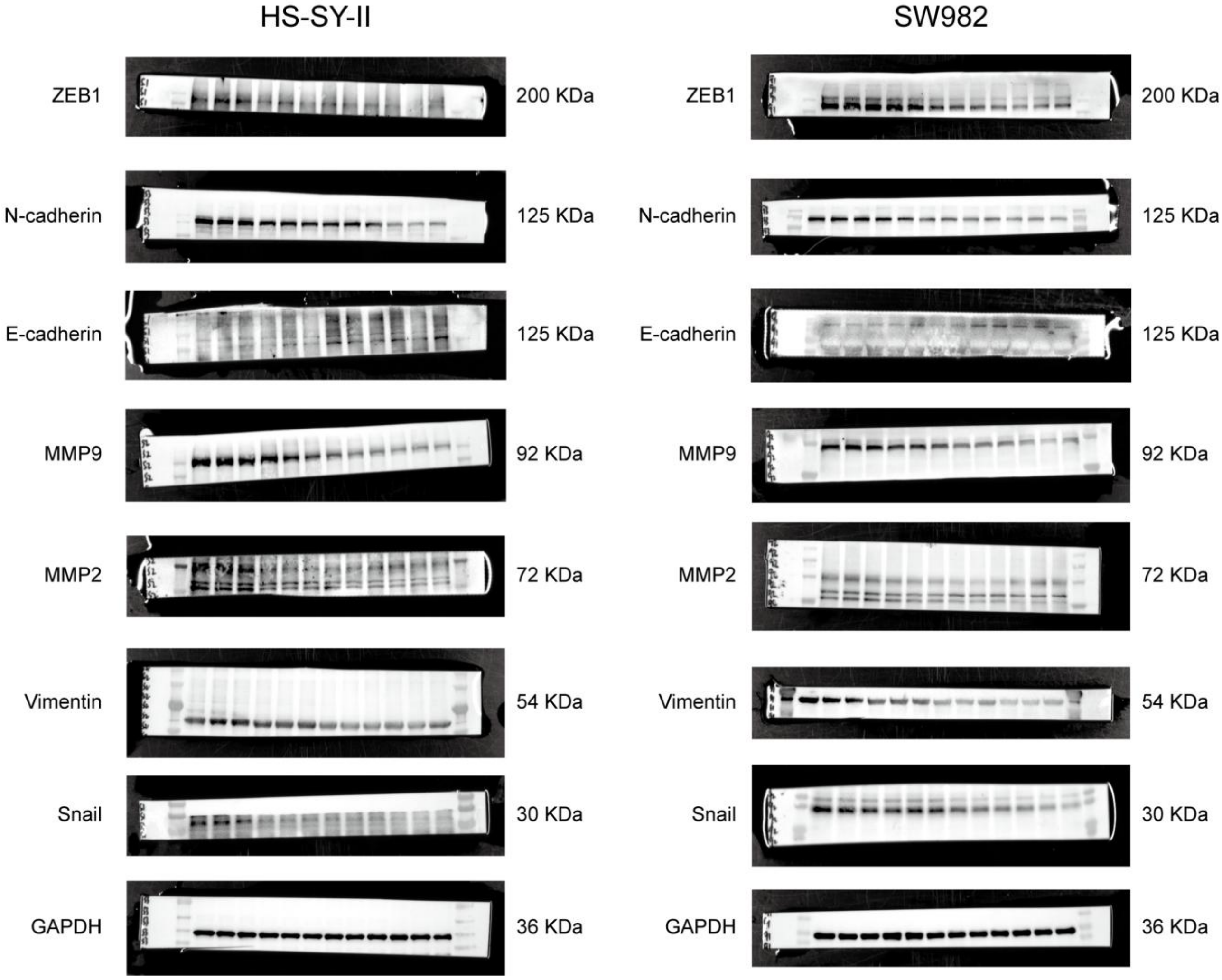

Figure 2H

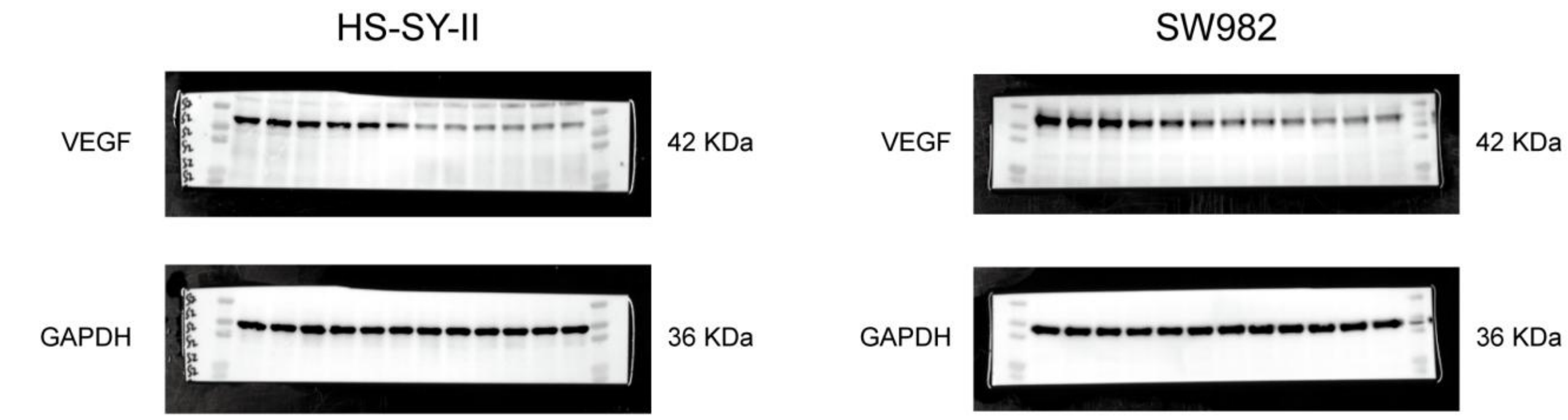

Figure 3D

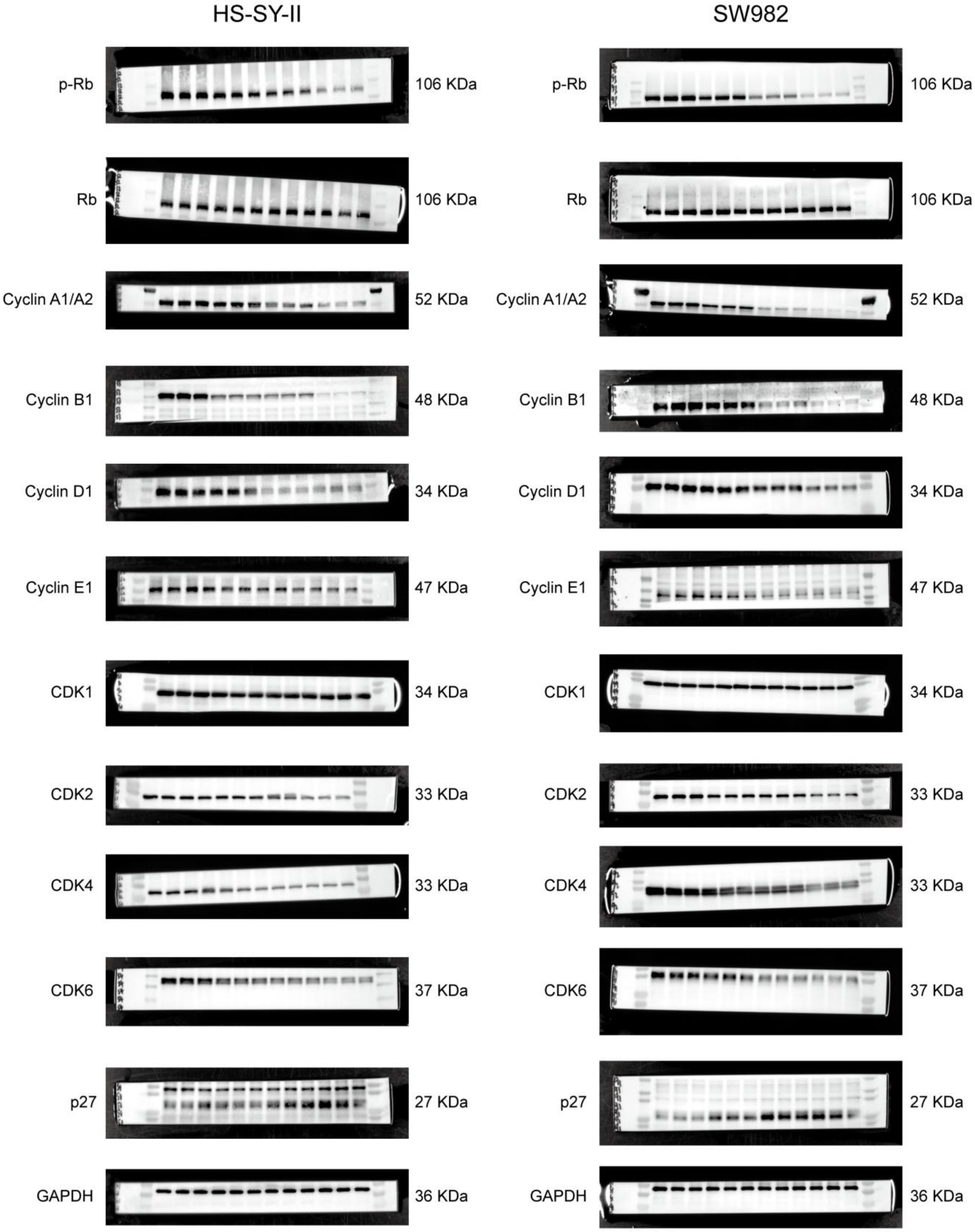
HS-SY-II
SW982
p-Rb
106 KDa
Rb
106 KDa
Cyclin A1/A2
52 KDa
Cyclin B1
48 KDa
Cyclin D1
34 KDa
Cyclin E1
47 KDa
CDK1
34 KDa
CDK2
33 KDa
CDK4
33 KDa
CDK6
37 KDa
p27
27 KDa
GAPDH
36 KDa

Figure 4B

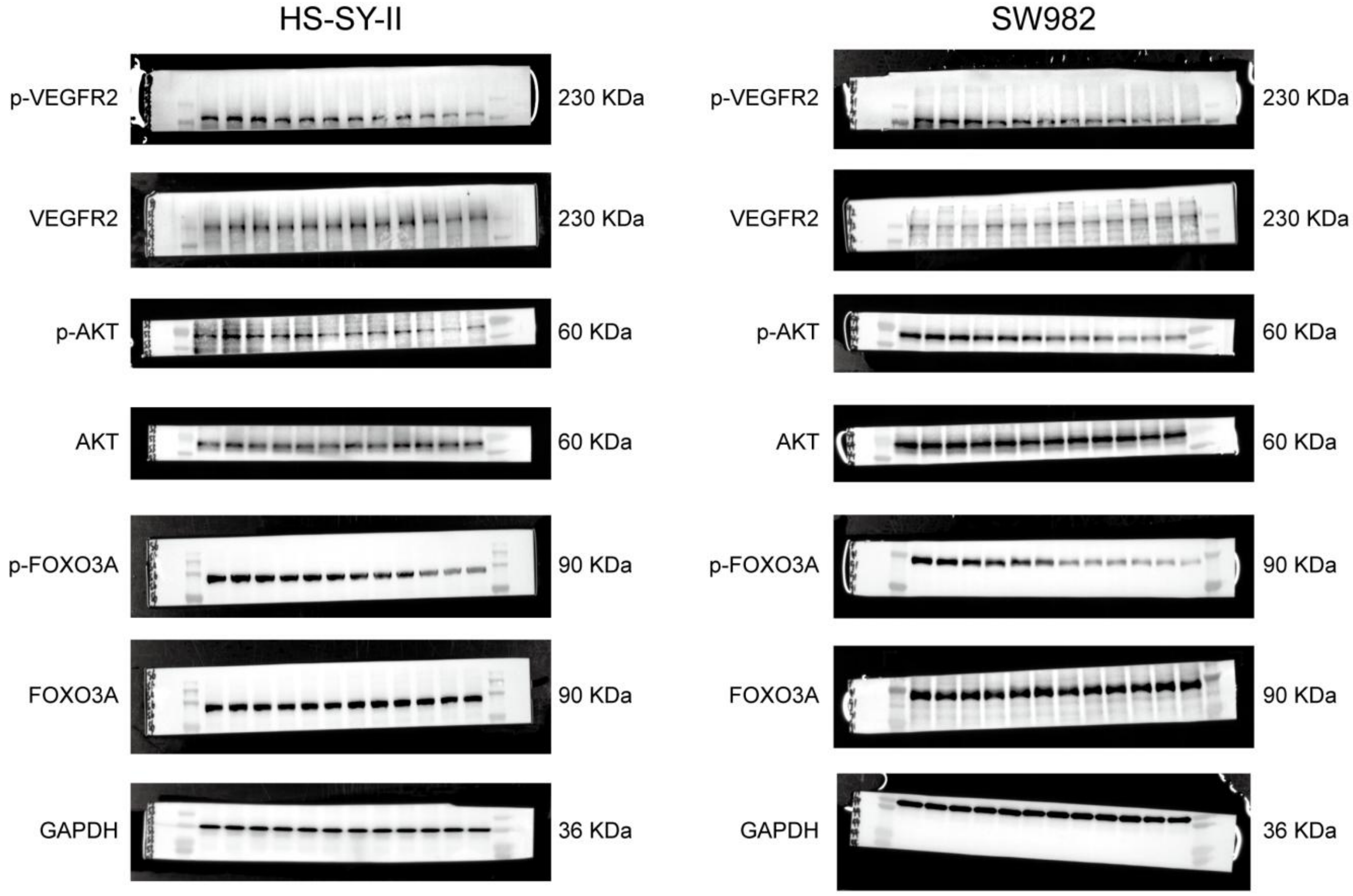

Figure 4C

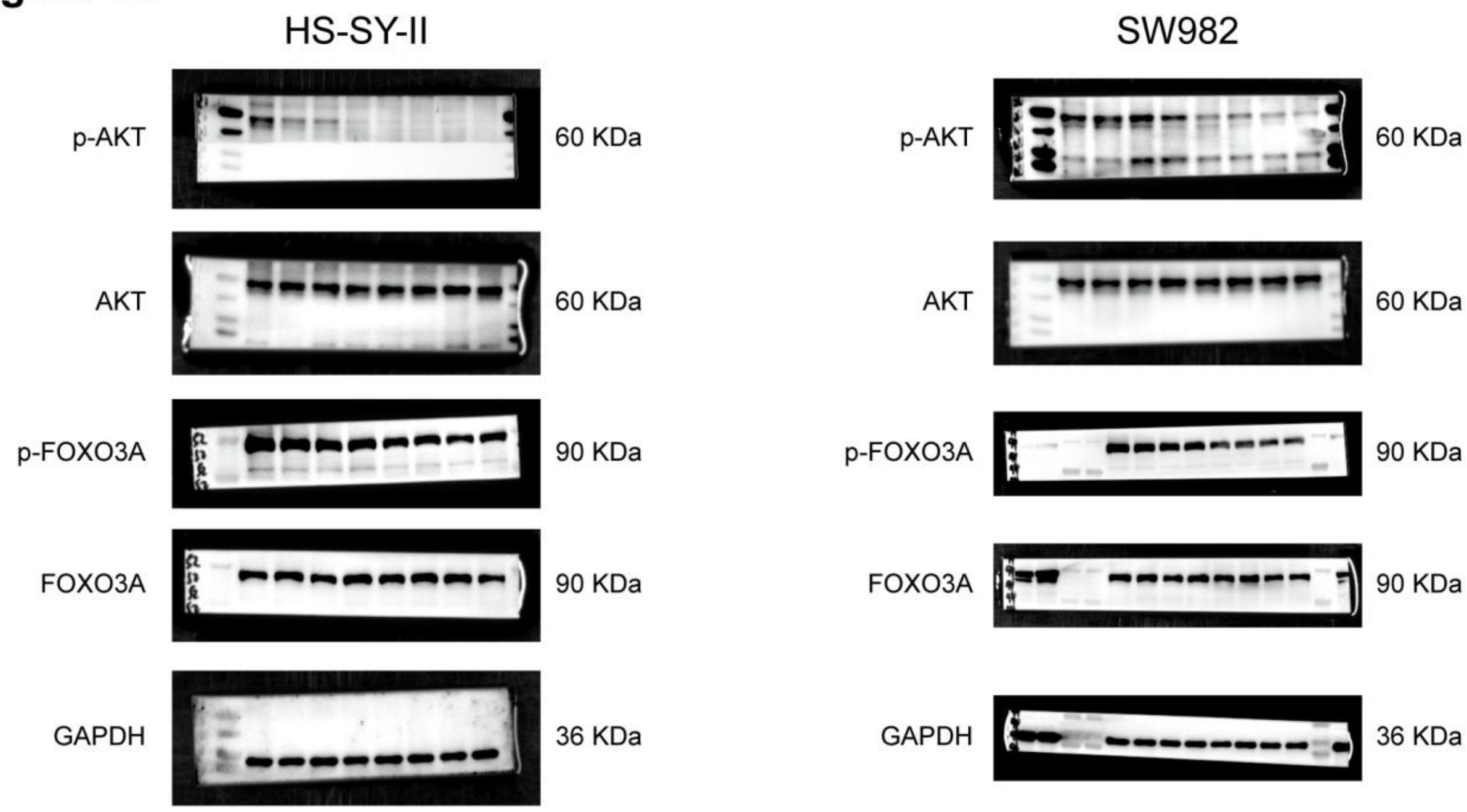

Figure 4D

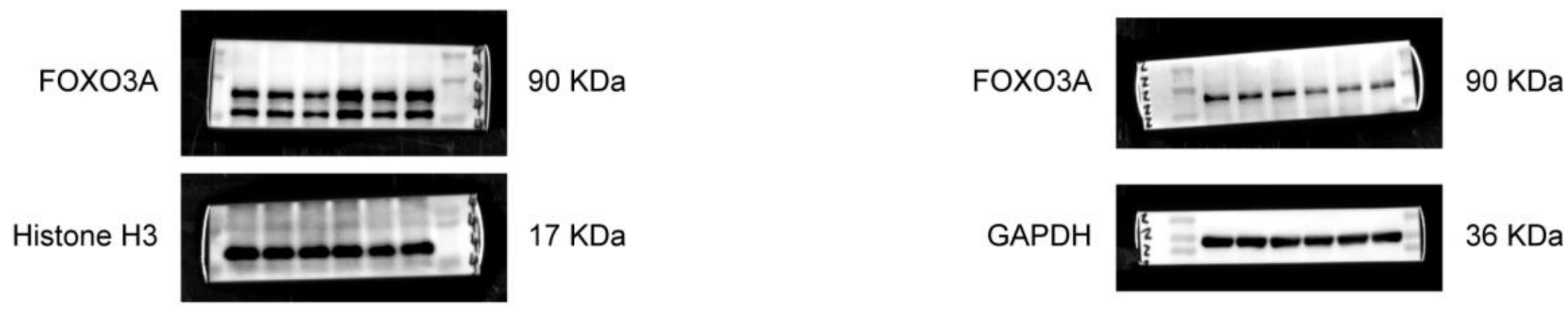

Figure 4F

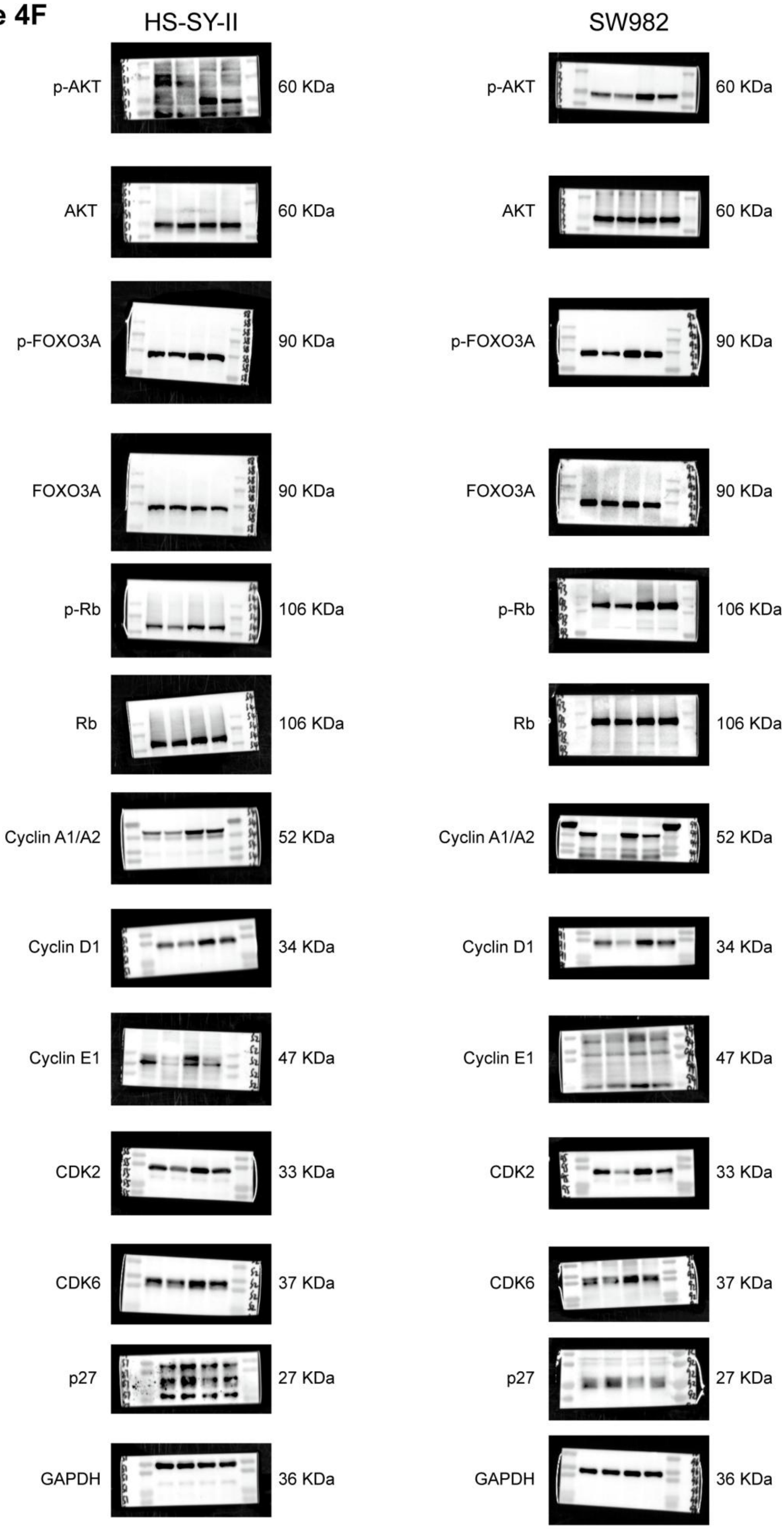
HS-SY-II
SW982
p-AKT
60 KDa
AKT
60 KDa
p-FOXO3A
90 KDa
FOXO3A
90 KDa
p-Rb
106 KDa
Rb
106 KDa
Cyclin A1/A2
52 KDa
Cyclin D1
34 KDa
Cyclin E1
47 KDa
CDK2
33 KDa
CDK6
37 KDa
p27
27 KDa
GAPDH
36 KDa

Figure 5A

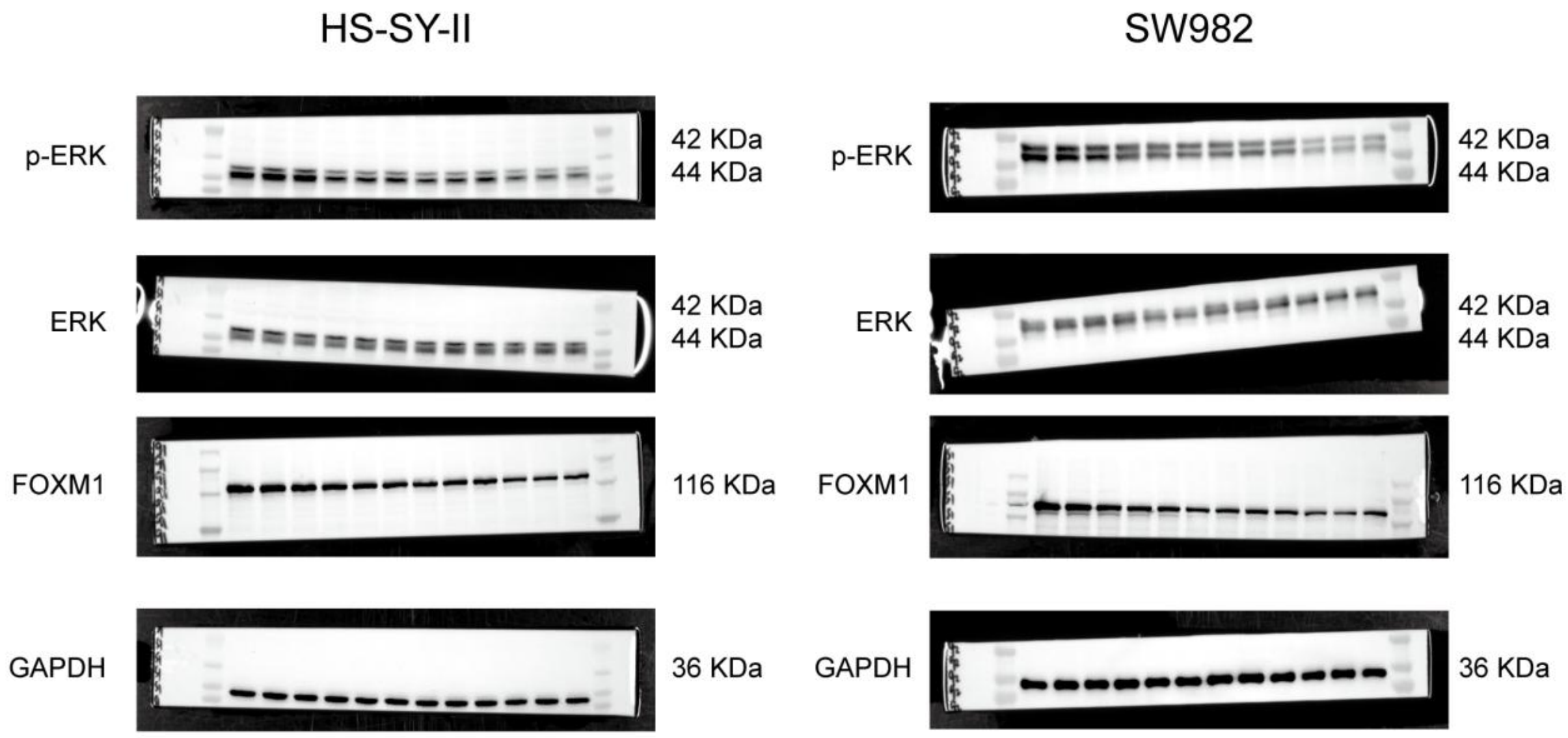

Figure 5B

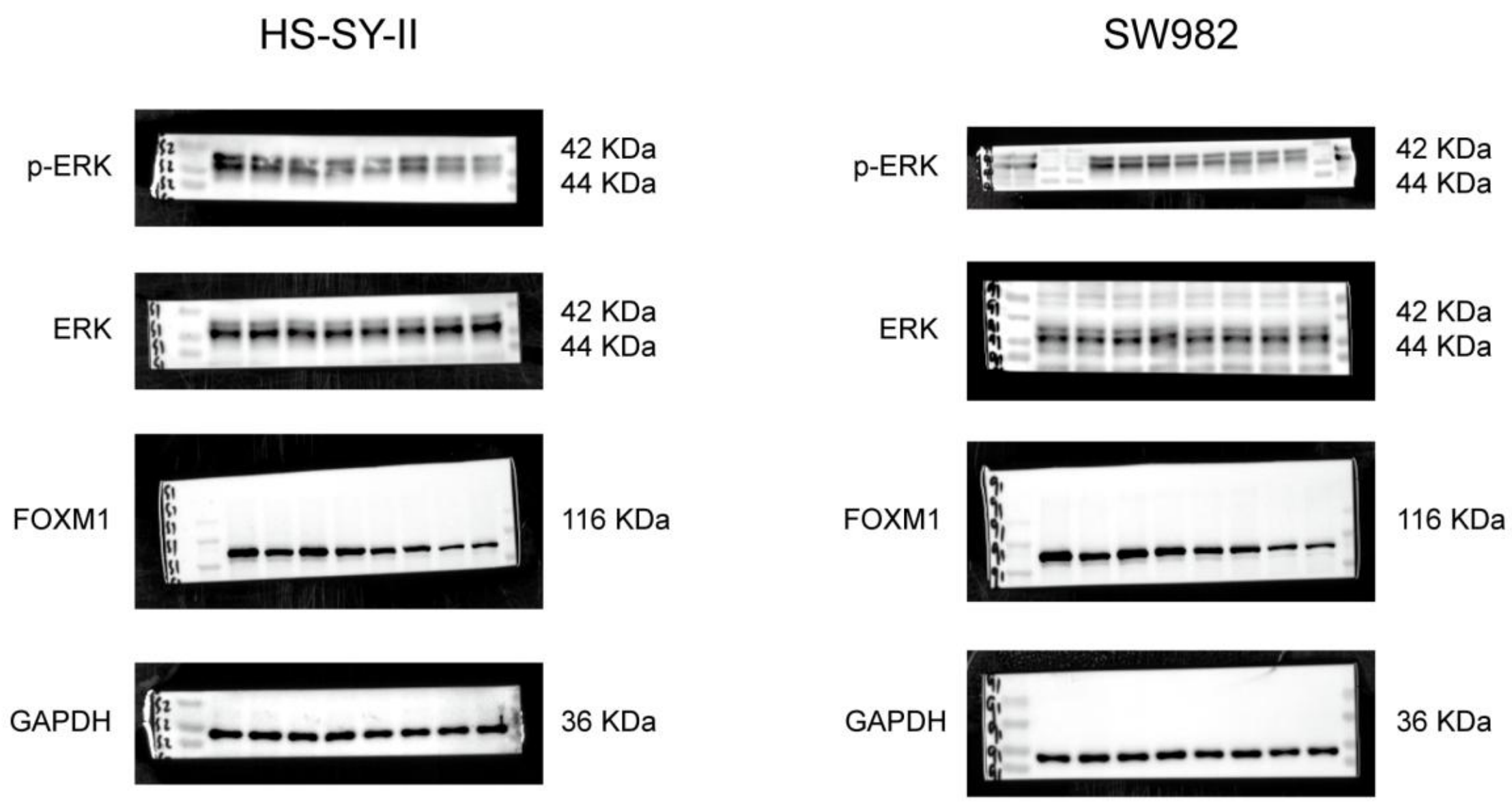

Figure 5E

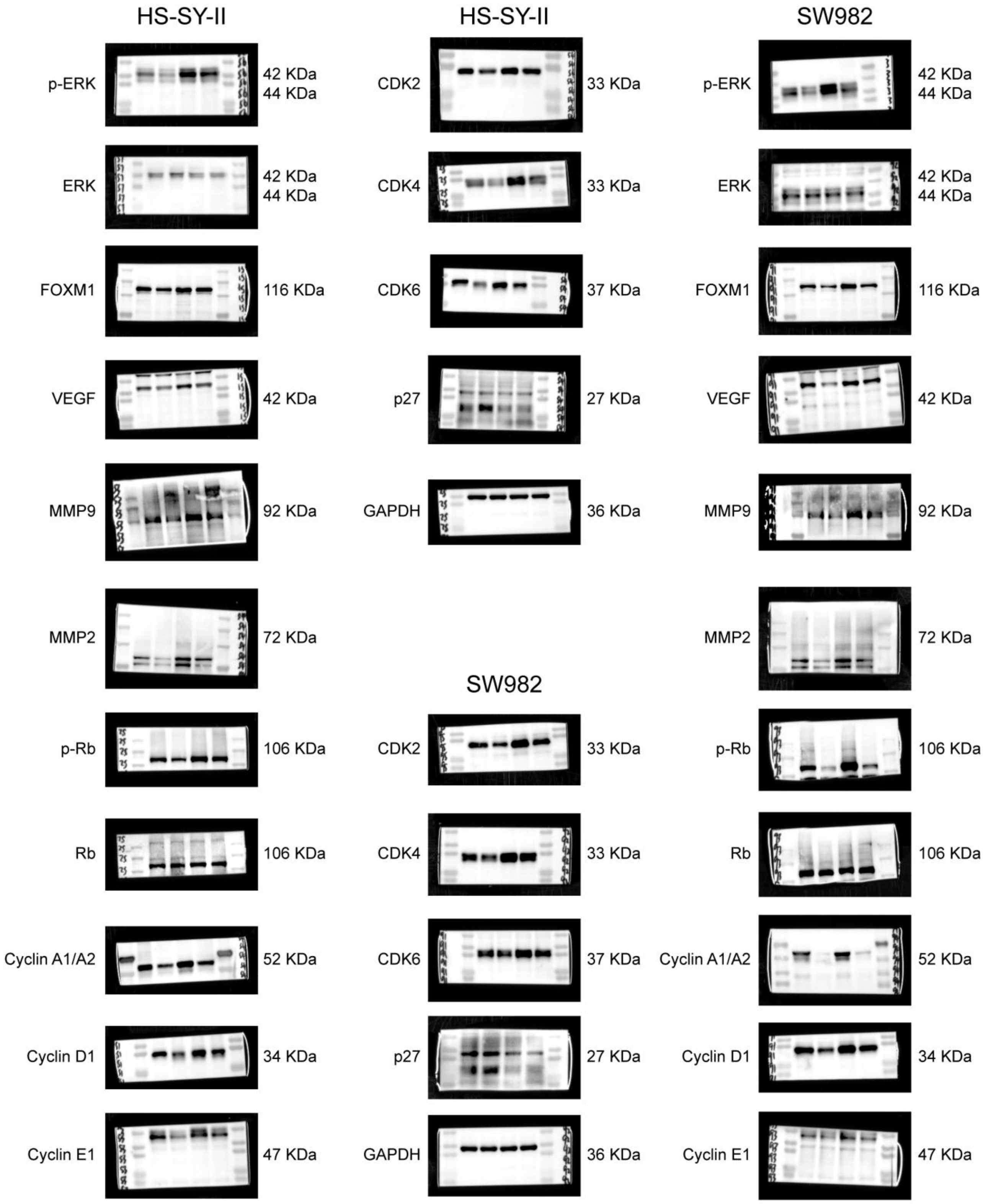

HS-SY-II
p-ERK
42 KDa
44 KDa
ERK
42 KDa
44 KDa
FOXM1
116 KDa
VEGF
42 KDa
MMP9
92 KDa
MMP2
72 KDa
p-Rb
106 KDa
Rb
106 KDa
Cyclin A1/A2
52 KDa
Cyclin D1
34 KDa
Cyclin E1
47 KDa
HS-SY-II
CDK2
33 KDa
CDK4
33 KDa
CDK6
37 KDa
p27
27 KDa
GAPDH
36 KDa
SW982
CDK2
33 KDa
CDK4
33 KDa
CDK6
37 KDa
p27
27 KDa
GAPDH
36 KDa
SW982
p-ERK
42 KDa
44 KDa
ERK
42 KDa
44 KDa
FOXM1
116 KDa
VEGF
42 KDa
MMP9
92 KDa
MMP2
72 KDa
p-Rb
106 KDa
Rb
106 KDa
Cyclin A1/A2
52 KDa
Cyclin D1
34 KDa
Cyclin E1
47 KDa

Figure 6C

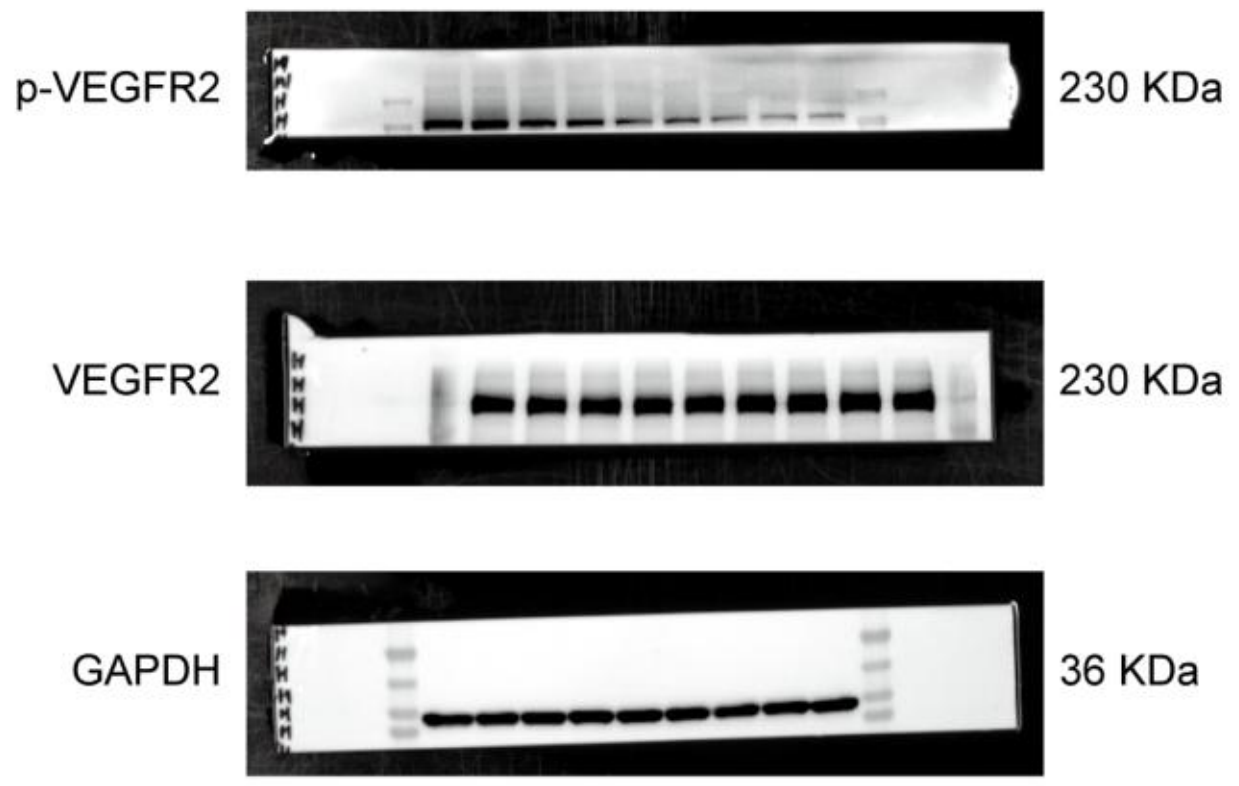

Figure 6D

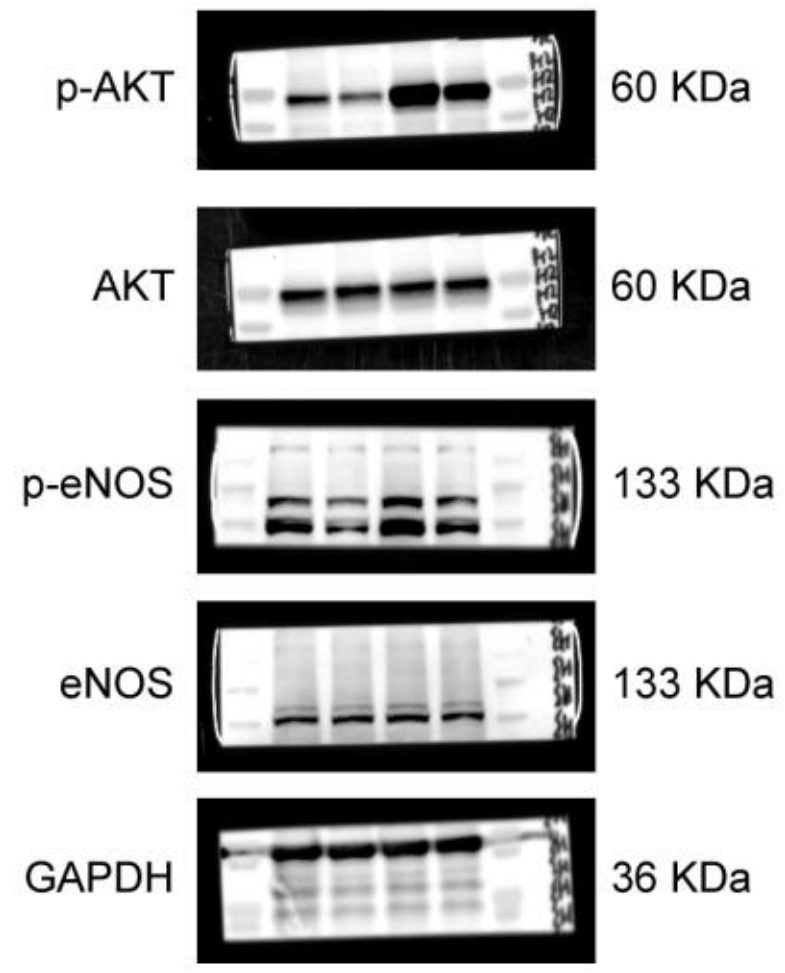

Figure 7E

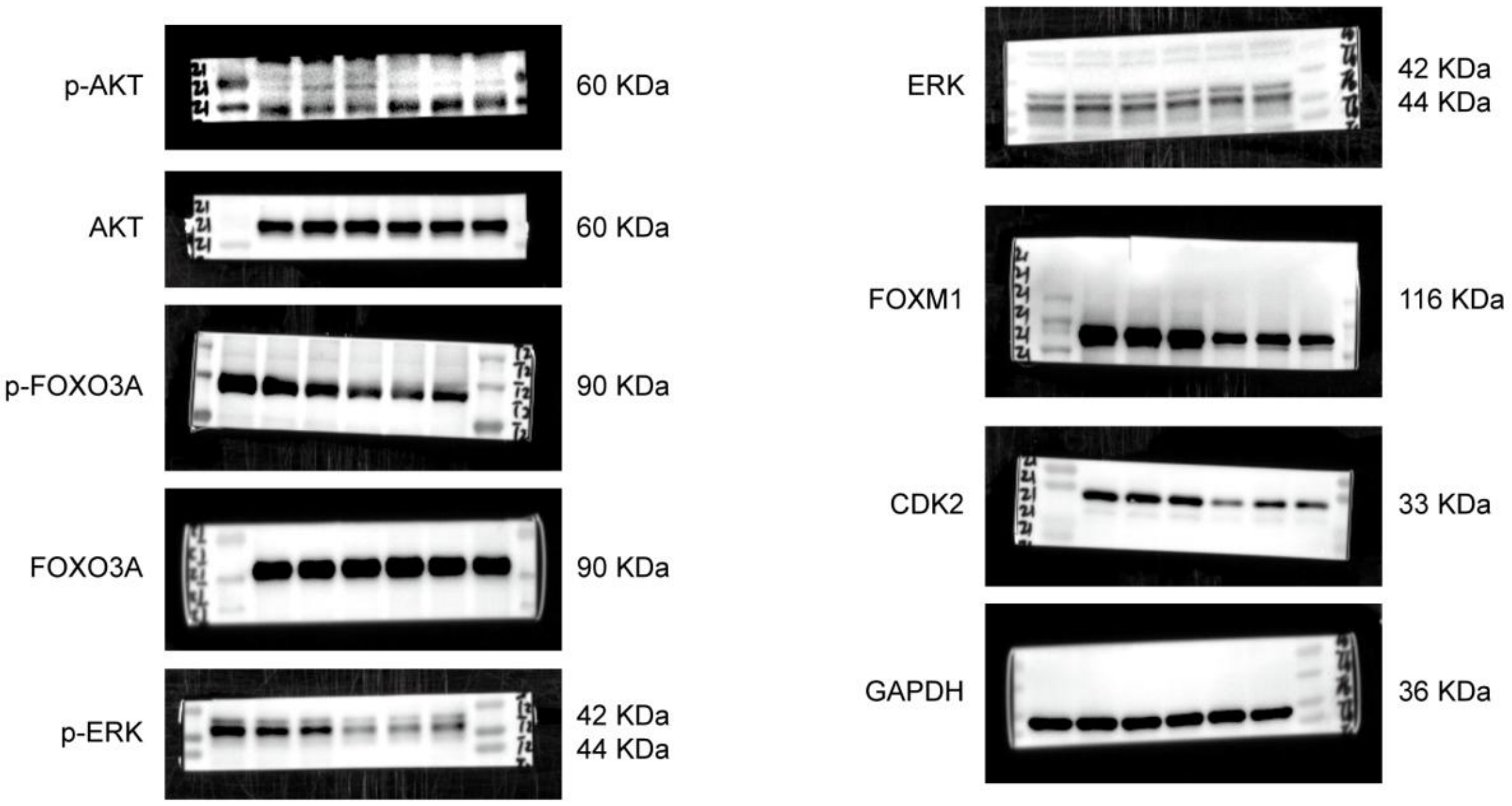

Figure S1A

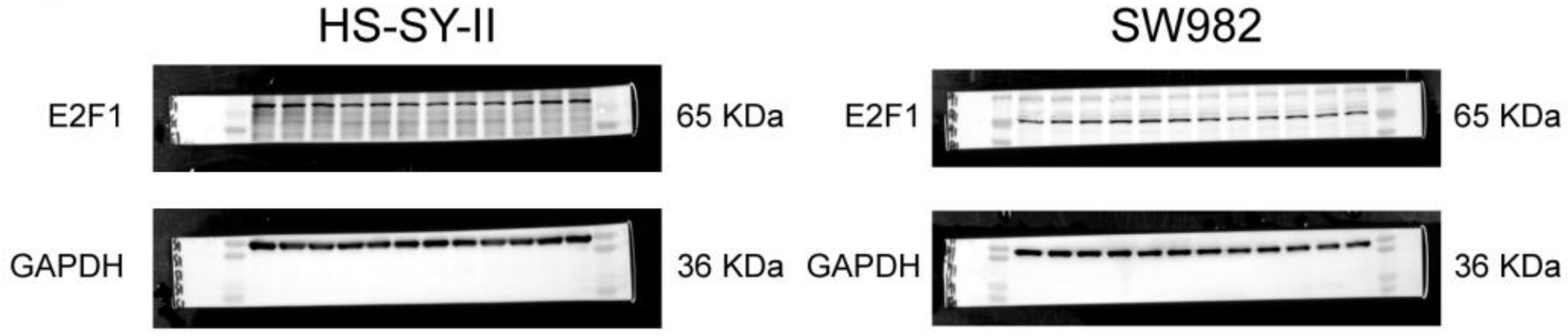

Figure S1C

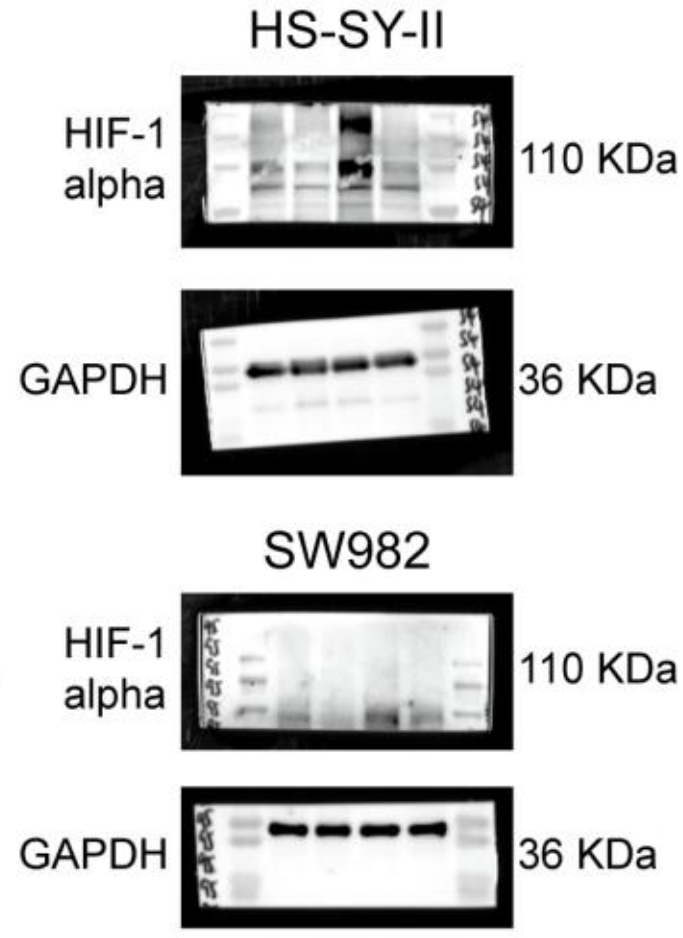

Figure S1B

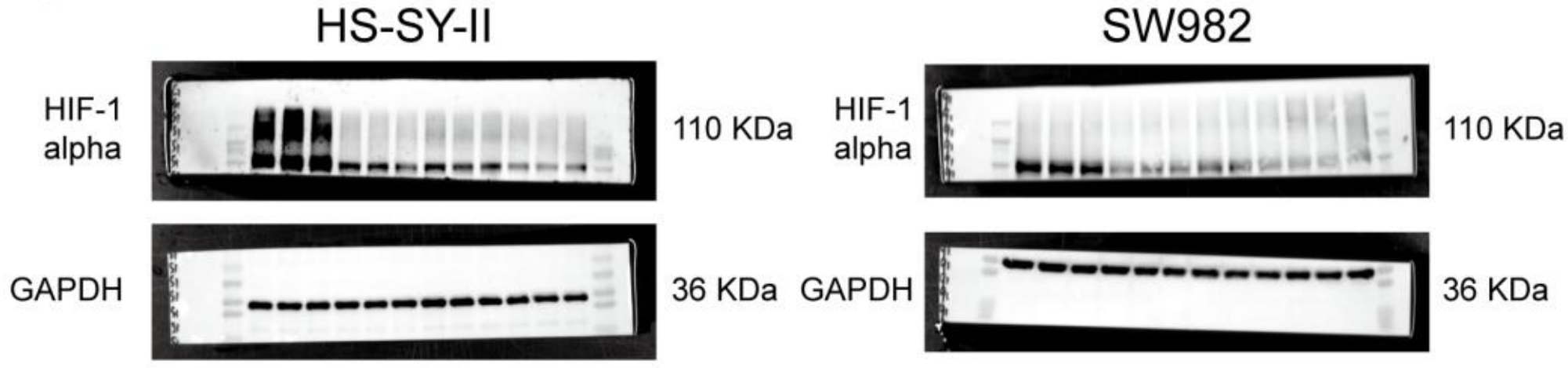

Supplement: Supplementary file 2 [file 41420_2026_3188_MOESM3_ESM.pdf]
