# Supplementary material for: Apatinib inhibits synovial sarcoma progression and angiogenesis via VEGFR2-mediated AKT/FOXO3A and ERK1/2/FOXM1 signaling pathways
Source: Cell Death Discov. 2026 Jun 3;12:328. doi: 10.1038/s41420-026-03188-7 (PMC13442848; doi:10.1038/s41420-026-03188-7)
Supplement: Supplementary file 3 [file 41420_2026_3188_MOESM2_ESM.docx]

**Supplementary Table 1. Antibodies used in the study.**

| **Antibody** | **Company** | **Catalogue No.** | **Application** | **Dilution** |
| --- | --- | --- | --- | --- |
| **Used for Western blotting (WB)** | | | | |
| anti-Caspase 3/P17/P19 | Proteintech | 19677-1-AP | WB | 1:1000 |
| anti-Bcl-2 | Proteintech | 12789-1-AP | WB | 1:2000 |
| anti-BAX | Proteintech | 50599-2-Ig | WB | 1:20000 |
| anti-Bcl-xL | Proteintech | 10783-1-AP | WB | 1:1000 |
| anti-LC3B | Abcam | ab192890 | WB | 1:2000 |
| anti-p62 | Abcam | ab207305 | WB | 1:1000 |
| anti-ATG5 | Epizyme | R013751 | WB | 1:1000 |
| anti-ZEB1 | Abways | CY7181 | WB | 1:1000 |
| anti-N-Cadherin | Abcam | Ab76011 | WB | 1:5000 |
| anti-E-Cadherin | Epizyme | R013244 | WB | 1:1000 |
| anti-MMP9 | Abways | CY5205 | WB | 1:1000 |
| anti-MMP2 | Abways | CY5189 | WB | 1:1000 |
| anti-Vimentin | Epizyme | M010697 | WB | 1:1000 |
| anti-Snail | Epizyme | R013727 | WB | 1:1000 |
| anti-VEGF | Boster | PB9071 | WB | 1:500 |
| anti-p-Rb | Epizyme | R015029 | WB | 1:1000 |
| anti-Rb | Epizyme | P013726 | WB | 1:1000 |
| anti-Cyclin A1/A2 | Epizyme | R011025 | WB | 1:1000 |
| anti-Cyclin B1 | Abways | CY5378 | WB | 1:1000 |
| anti-Cyclin D1 | Epizyme | P012243 | WB | 1:1000 |
| anti-Cyclin E1 | Epizyme | R013802 | WB | 1:1000 |
| anti-CDK1 | Abways | CY5176 | WB | 1:1000 |
| anti-CDK2 | Epizyme | R011804 | WB | 1:1000 |
| anti-CDK4 | Epizyme | R012806 | WB | 1:1000 |
| anti-CDK6 | Epizyme | R013385 | WB | 1:1000 |
| anti-p27 | Abways | CY5354 | WB | 1:1000 |
| anti-E2F1 | Abways | CY6580 | WB | 1:1000 |
| anti-p-VEGFR2 | Affinity | AF4426 | WB | 1:1000 |
| anti-VEGFR2 | Proteintech | 26415-1-AP | WB | 1:1000 |
| anti-p-AKT | CST | 4058S | WB | 1:1000 |
| anti-AKT | CST | 4691S | WB | 1:1000 |
| anti-p-FOXO3A | Abways | CY5562 | WB | 1:1000 |
| anti-FOXO3A | Abways | CY5079 | WB | 1:1000 |
| anti-HIF-1 alpha | Epizyme | R015076 | WB | 1:1000 |
| anti-p-Erk1/2 | CST | 9101S | WB | 1:1000 |
| anti-Erk1/2 | CST | 9102S | WB | 1:1000 |
| anti-FOXM1 | Santa Cruz | sc-376471 | WB | 1:500 |
| anti-p-eNOS | Affinity | AF3247 | WB | 1:1000 |
| anti-eNOS | Abways | CY3412 | WB | 1:500 |
| HRP Goat Anti-Rabbit IgG | Abways | AB0101 | WB | 1:5000 |
| HRP Goat Anti-Mouse IgG | Abways | AB0102 | WB | 1:5000 |
| **Used for Immunofluorescence (IF) and immunohistochemical (IHC) staining** | | | | |
| Anti-Cleaved-Caspase 9 | Abways | CY5682 | IF | 1:50 |
| anti-Vimentin | Epizyme | M010697 | IF | 1:100 |
| anti-Snail | Epizyme | R013727 | IF | 1:100 |
| anti-p-AKT | CST | 4058S | IF | 1:100 |
| anti-FOXM1 | Santa Cruz | sc-376471 | IF | 1:100 |
| anti-p-VEGFR2 | Affinity | AF4426 | IHC | 1:100 |
| anti-p-AKT | Huabio | ET1607-73 | IHC | 1:800 |
| anti-p-FOXO3A | Abways | CY5562 | IHC | 1:200 |
| anti-FOXM1 | Santa Cruz | sc-376471 | IHC | 1:200 |
| anti-CDK2 | Epizyme | R011804 | IHC | 1:200 |
